# Oxidation of [3]naphthylenes to cations and dications converts local paratropicity into global diatropicity

**DOI:** 10.3762/bjoc.21.20

**Published:** 2025-02-05

**Authors:** Abel Cárdenas, Zexin Jin, Yong Ni, Jishan Wu, Yan Xia, Francisco Javier Ramírez, Juan Casado

**Affiliations:** 1 Department of Physical Chemistry, University of Málaga, Andalucia-Tech, Campus de Teatinos s/n, 29071 Málaga, Spain; 2 Department of Chemistry, Stanford University, Stanford, CA 94305, USA; 3 Department of Chemistry, National University of Singapore, 3 Science Drive 3, Singapore, Singapore

**Keywords:** ACID, aromaticity, force constants, NICS, spectroscopy

## Abstract

Oxidized states of polycyclic aromatic hydrocarbons are of importance as they represent charged conductive species in organic semiconductor substrates. In this work, we investigated the properties of radical cations and dications of linear and angular [3]naphthylenes, consisting of fused aromatic naphthalenoid and antiaromatic cyclobutadienoid moieties and containing different degrees of paratropicity. Electronic absorption and vibrational Raman spectroscopies were used to describe the more relevant bonding changes. Stretching force constants were evaluated to monitor the aromatic–antiaromatic alternation pattern upon oxidation. They showed us that the dication of linear [3]naphthylene became an overall global π-electron delocalized molecule. This result was supported by nucleus independent chemical shift (NICS) calculations and anisotropy of the current induced density (ACID) plots, as they evidenced the presence of a perimetral diatropic global ring current upon oxidation.

## Introduction

Since the discovery of conjugated polymers, it has been very insightful to study the molecular transformations associated with the generation of cationic species in conjugated aromatic oligomers displaying one-dimensional π-electron delocalization [1]. Oligothiophenes [2] and oligo(*para*-phenylene vinylenes) [3] have been used as models of systems in which charge defects are responsible for conductivity in their corresponding conducting polymers. Acenes are the archetypal structure of small polycyclic aromatic hydrocarbons with a π-electron structure expanded over a sequence of linearly fused benzenes [4]. Whereas acenes, up to substituted pentacenes, are relatively stable molecules under ambient conditions, longer acenes undergo spontaneous dimerization and react with oxygen [5–6], owing to the rising diradical character. This behavior was also observed in the oxidized species of shorter acenes [7]. Surprisingly, Bettinger and Einholz [5] reported a stable heptacene dication in concentrated sulfuric acid, a stability attributed to the intermolecular Coulomb repulsion between the charged molecules, which prevents the dimerization of the acene. This exciting finding suggests possible modes of kinetic stabilization of oxidized species of π-conjugated compounds that are unstable in their neutral ground-electronic states.

**Figure 1:**
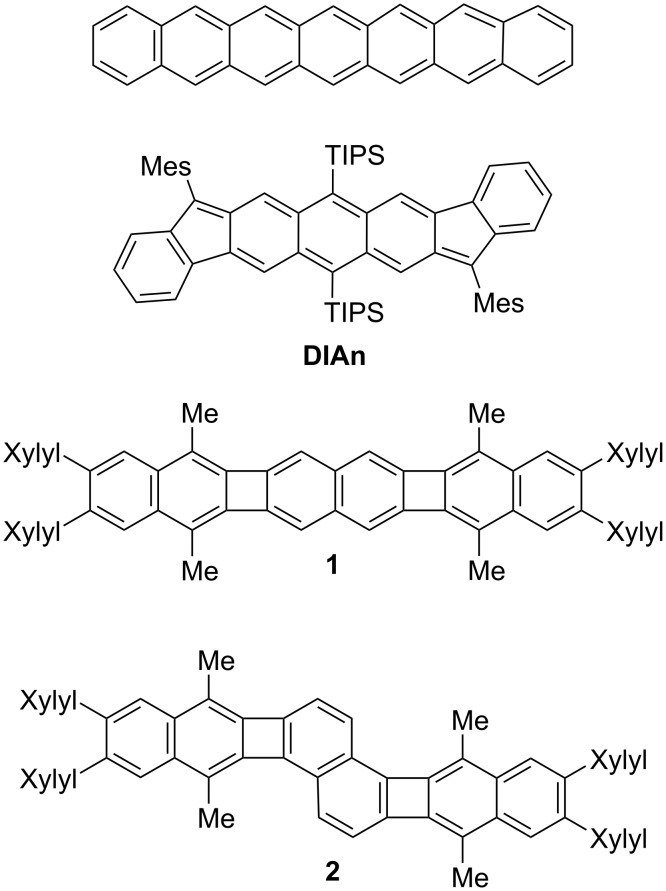
Chemical structures of heptacene, diindenoanthracene (**DIAn**), and the molecules of **1** and **2** studied in this work (TIPS: triisopropylsilyl, Mes: mesityl, Me: methyl).

According to the Hückel theory, antiaromatic molecules contain 4*n* π-electrons (*n* = 1, 2, 3…) and are highly unstable [8]. Though the antiaromatic molecules are much less common than their aromatic counterparts, they have attracted a growing interest in recent times, both from fundamental and technological reasons [9–11]. Antiaromaticity destabilizes the ground state of organic molecules by raising their highest energy occupied molecular orbitals, thus allowing for easy oxidation, doping, and electron-transfer reactions, all of which lead to conductive and photoactive species [9,12]. Given the inherent instability of neutral antiaromatic systems, including those systems containing fragments or moieties with local antiaromaticity, the detailed structural properties of the charged species formed from neutral antiaromatic precursors remain challenging to study. Haley and some of us reported the oxidization of partially antiaromatic diindenoanthracene, **DIAn**, Figure 1, forming charged molecules stabilized by the rearomatization of the central anthracene unit [13–14]. Porphyrinoid-based molecules [15–17] have also been reported as model systems to investigate redox charged species experiencing evolutions from neutral non-aromatic, to antiaromatic, and to aromatic structures.

Xia and co-workers recently reported a modular method to synthesize molecules containing cyclobutadienoid (CBD) groups [18–20], including [3]naphthylenes **1** and **2** in Figure 1 [18]. They are endowed by three aromatic naphthalenoid (NAP) moieties, fused by two antiaromatic CBD ones in two different topologies. Structurally, these polycyclic π-conjugated hydrocarbons consist of eight fused rings and thirty π-electrons. In this work, we report that compounds **1** and **2** can both be easily oxidized to form relatively stable radical cations (**1^•+^**, **2^•+^**) and dications (**1^2+^**, **2^2+^**). Interestingly, oxidation reverses local antiaromaticity to aromaticity, a transition that is particularly noticeable in **1 → 1^•+ ^→ 1^2+^**, where stabilization of the dication is associated to the appearance of a global diatropic ring current which stabilizes the whole molecule. On the contrary, **2^2+^** can be better viewed as two segregated radical cations with slight, but high enough, local diatropic character in each. Here, we use electronic UV–vis–NIR absorption and vibrational Raman spectroscopies, normal mode and force field calculations, and magnetic-based analysis to gain comprehensive understanding of the electronic and molecular structures of the oxidized forms of these aromatic/antiaromatic molecular amalgams, aiming to discover the driving forces that govern the stabilization of such redox states.

## Results and Discussion

### Electrochemistry

**Figure 2:**
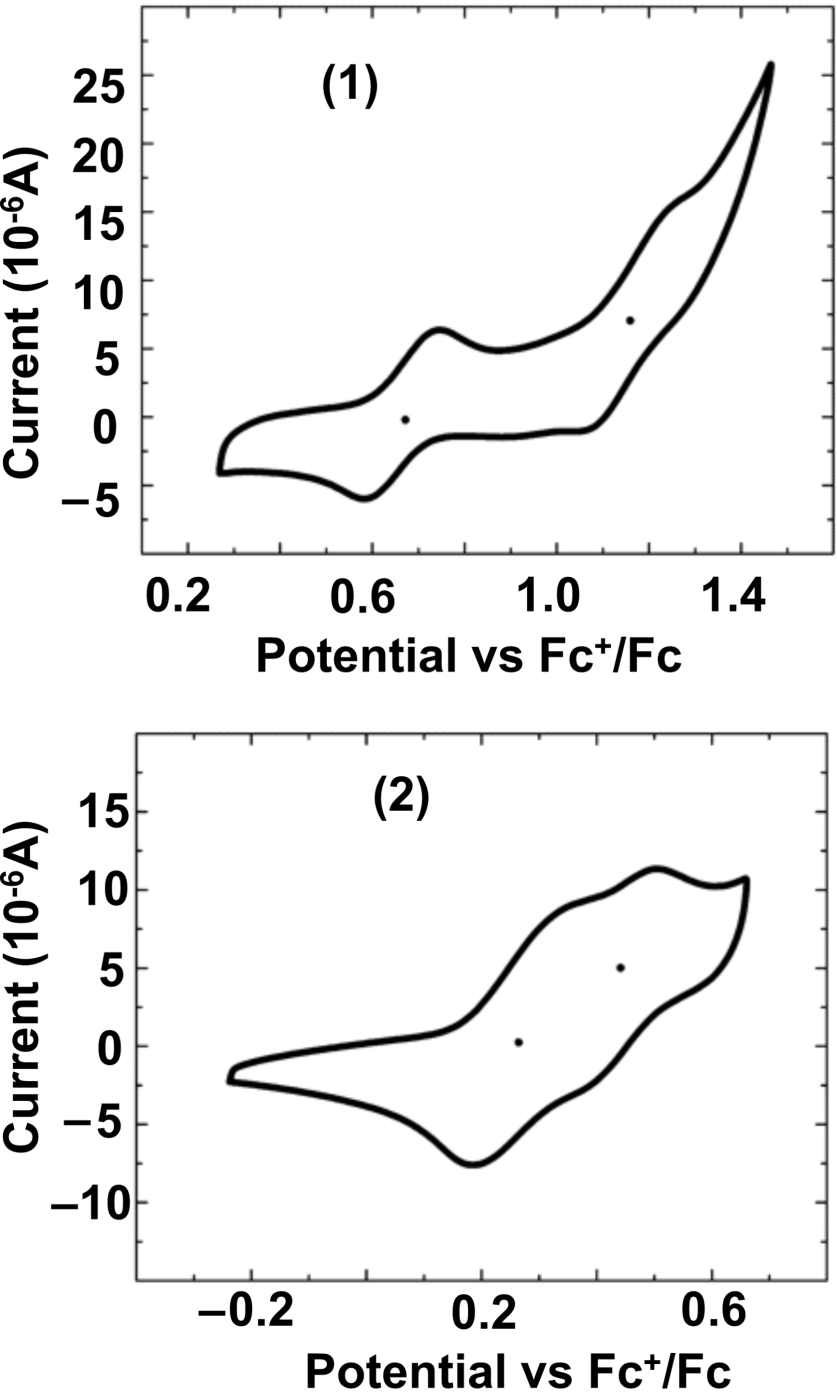
Cyclic voltammograms of **1** and **2**.

Figure 2 shows the electrochemical cyclic voltammograms of **1** and **2**, in which two reversible oxidation processes can be observed. By considering the half-wave potential values obtained from the cyclic voltammograms, two one-electron peaks, at 0.67 and 1.16 V, were clearly resolved for the linear oligomer **1**. For the angular molecule **2**, two partially overlapped one-electron peaks appeared at noticeably lower voltages, 0.28 and 0.44 V. This result reflects the higher energy lying HOMO (and easy of oxidation) and stronger overall antiaromatic character of **2** compared to **1**. The decrease of the oxidation potentials with increasing antiaromaticity in the neutral states is opposite to the case of aromatic oligomers [21], where molecules with greater aromatic character have higher oxidation potentials. In addition, in **1^•+^** and **1^2+^** the charge defects are extended over the whole molecule (vide infra). Thus, an extended π-electron delocalization effect in **1^•+^** stabilizes the cation and shifts anodically the second oxidation.

On the contrary, the charge in **2^•+^** is expected to be largely confined in the central NAP, and the second oxidation would give rise to two naphthylene-centered cations located at both sides of the molecule. The connection path would be partially interrupted by the angular topology, thus accounting for the more similar redox potentials.

### Electronic spectroscopy

**Figure 3:**
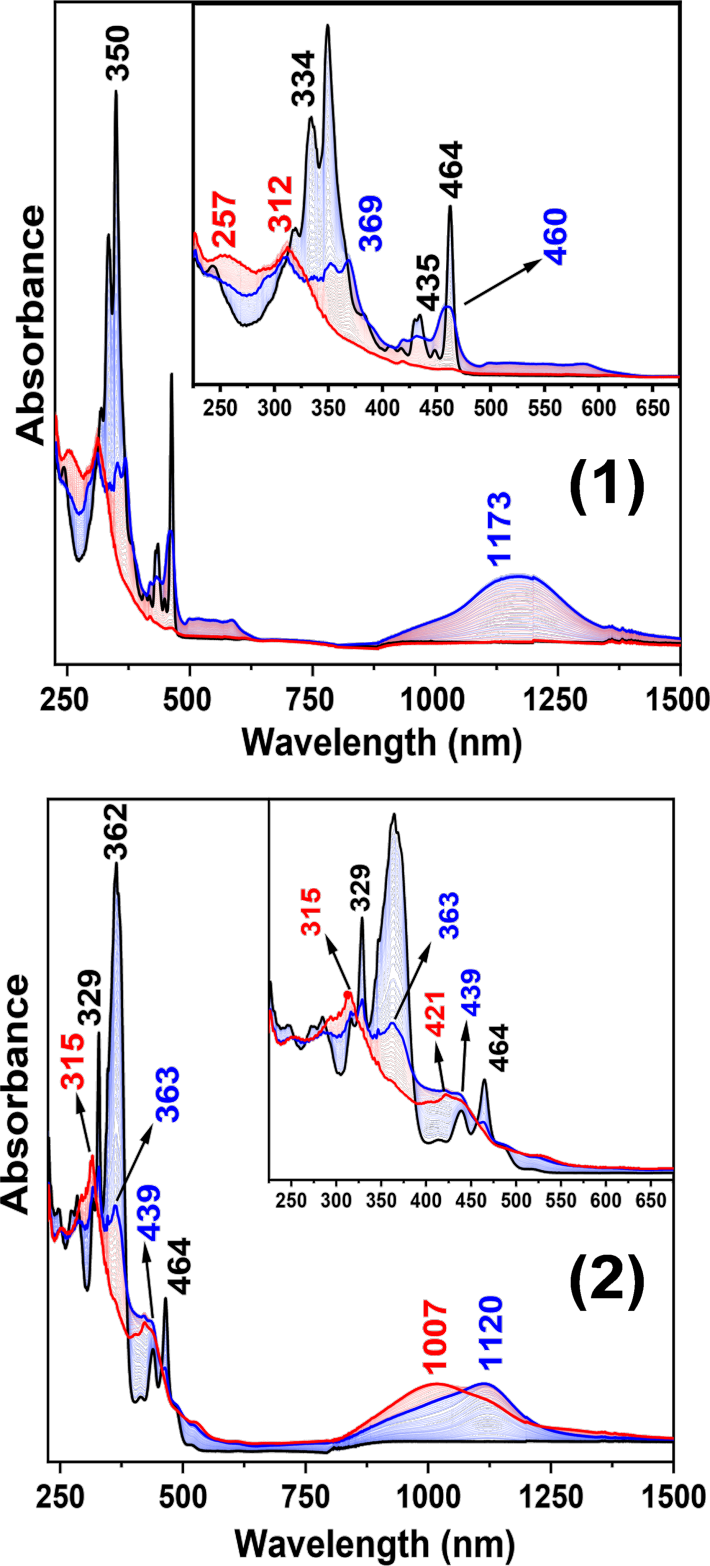
UV–vis–NIR electronic absorption spectra of **1** (top) and **2** (bottom) during the electrochemical oxidation in 0.1 M *n*-Bu_4_N·PF_6_ in CH_2_Cl_2_ at room temperature. The traces are black lines for neutral, blue lines for radical cation, and red lines for dication species.

The UV–vis–NIR electronic absorption spectra of the neutral and oxidized species of compounds **1** and **2** are shown in Figure 3. Initial electrochemical oxidation of **1** resulted in the progressive replacement of its absorption bands by three new features, which were assigned to the **1^•+^** radical cation, namely at 352/369 nm, a multiplet in the 500–600 nm interval, and a broad peak centered at 1173 nm. Further oxidation resulted in a quite silent vis–NIR spectrum characterized by one main peak at 312 nm, which was assigned to the **1^2+^** dication. The spectrum of the first oxidized species of **2**, the radical cation **2^•+^**, shows a band at 363 nm, a shoulder at 439 nm, and a broad absorption at 1120 nm. Nonetheless, the spectra of the dications **1^2+^** and **2^2+^** display noticeably differences given that the features at 421 and at 1007 nm, present in the angular [3]naphthylene **2,** are apparently absent in the linear isomer **1**.

### Raman spectroscopy

**Figure 4:**
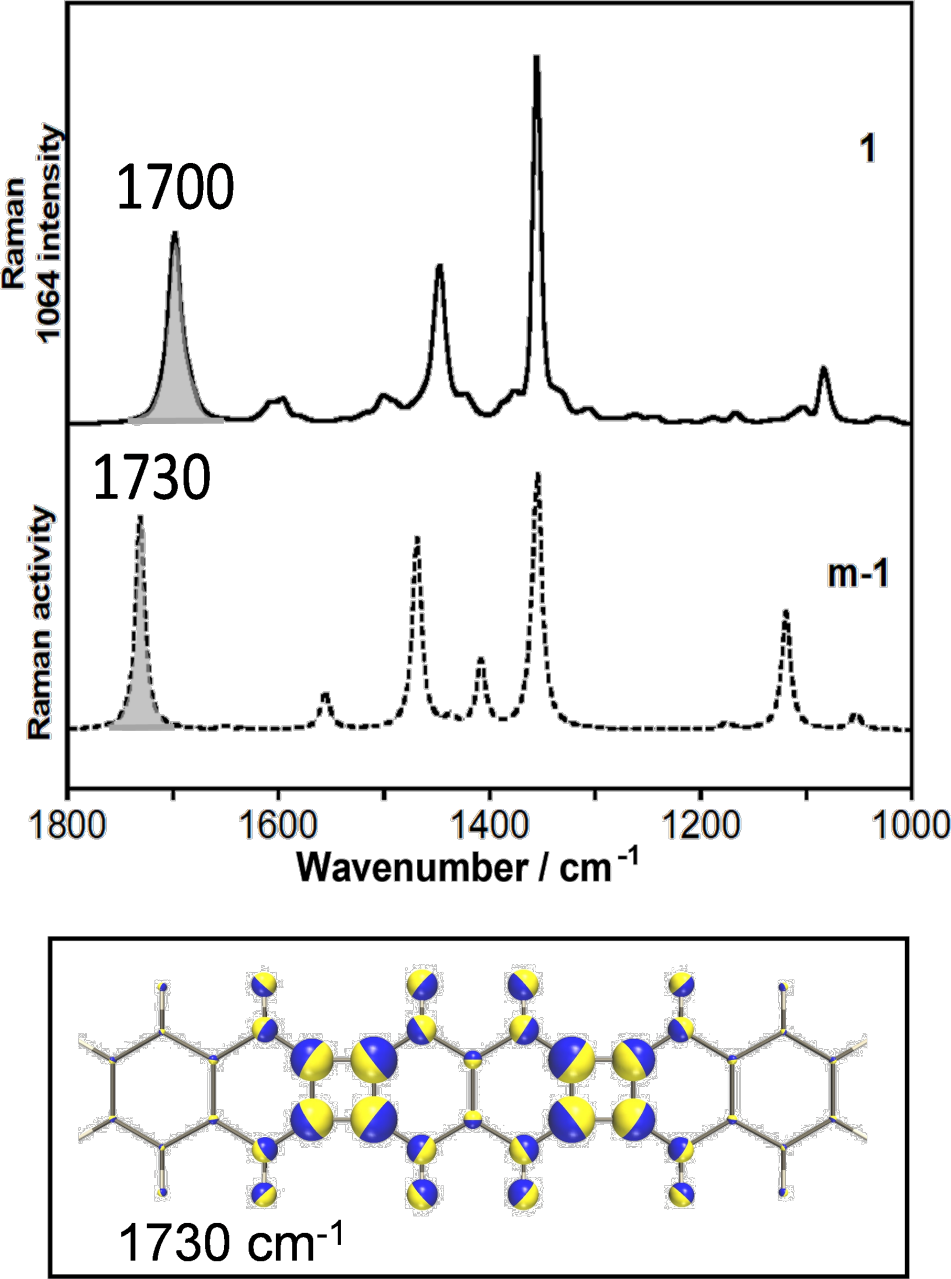
Top: B3LYP/6-311G(d,p) theoretical Raman spectrum of an unsubstituted model of **1** (denoted as **m-1** dotted lines which is **1** without methyl nor xylyl groups) compared with the experimental FT-Raman spectrum of **1**. Bottom: zoom in the CBO moiety of the vibrational normal mode associated with the theoretical Raman band at 1730 cm^−1^.

Figure 4 shows the experimental Raman spectrum of **1** in the neutral state and the theoretical Raman spectrum of a model molecule, **m-1** (**1** without the xylyl and methyl substituents, Figure S1 in Supporting Information File 1), which can be closely correlated to the spectrum of **1**. In Tables S1–S6 of Supporting Information File 1, the theoretical characterization of all molecules is presented. A detailed assignment of the most relevant Raman bands obtained after vibrational analysis from the theoretical Raman spectrum obtained for the neutral state of **m-1** is included in Table S7 of Supporting Information File 1. The most significant Raman feature for the neutral species was measured at 1700 cm^−1^ and was predicted at 1730 cm^−1^ for **m-1**. This band was assigned to the symmetric CC stretching mode of the four bonds that are exocyclic to CBD, ν(CC)_exo-CBD_, or CBD breathing mode, as indicated by the atomic displacements shown in Figure 4. Other two strong Raman features were measured at 1448 and 1355 cm^−1^. They were predicted at 1468 and 1354 cm^−1^, respectively, for **m-1**, being assigned to CC stretching modes with slight contributions of aromatic CH bending vibrations. The complete eigenvector for these vibrations can be seen in Figure S2 in Supporting Information File 1. Given that former normal mode mainly involves the CBD moieties, the observed feature at 1700 cm^−1^ can be considered a suitable marker band of the structural and electronic changes of the antiaromatic CBD rings upon oxidation.

**Figure 5:**
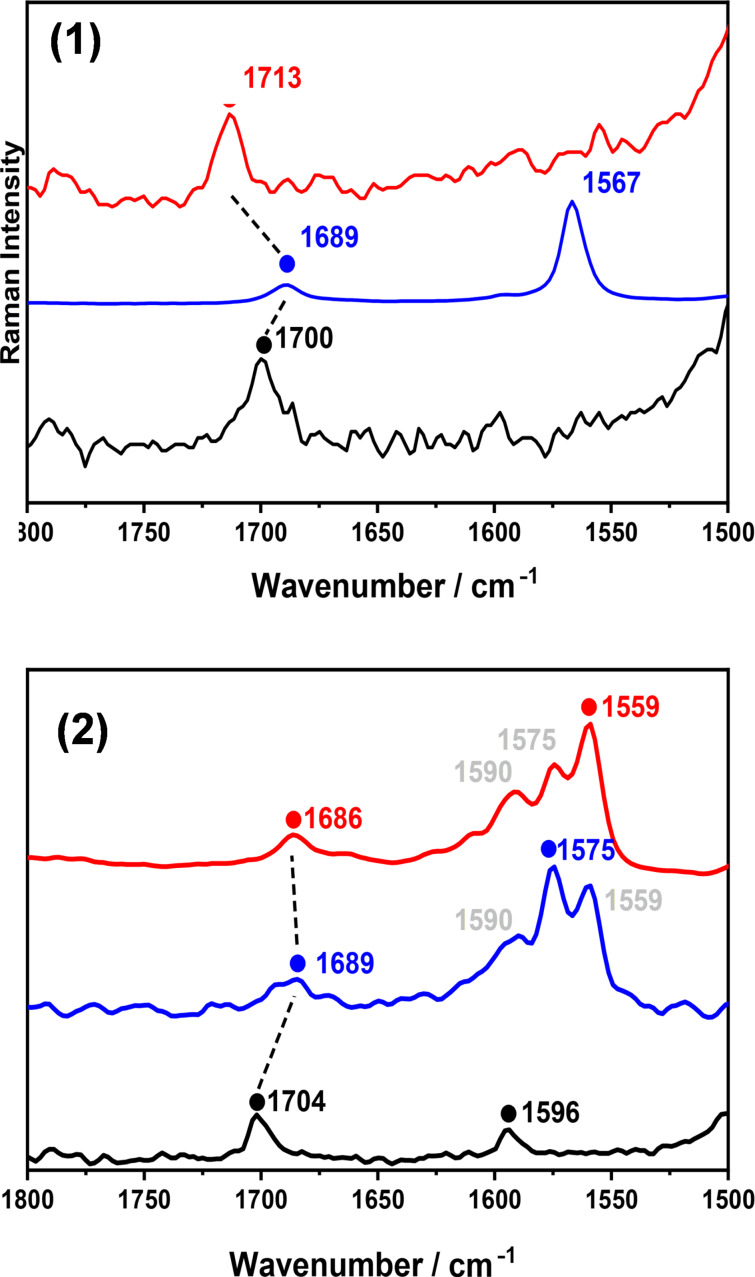
FT-Raman spectra in CH_2_Cl_2_ (approx. 10^−2^ to 10^−3^ M) of: top) **1** (black), **1^•+^** (blue), and **1^2+^** (red). Bottom) **2** (black), **2^•+^** (blue), and **2^2+^** (red). Oxidations are carried out by stepwise addition of NOBF_4_ in CH_2_Cl_2_.

The Raman spectra of **1**, **1^•+^**, and **1^2+^** are shown in Figure 5. Relative to **1**, the spectrum of **1^•+^** is characterized by a downshift by 11 cm^−1^ of the ν(CC)_exo-CBD_ vibration, together with the emergence of a new band at 1567 cm^−1^ which was assigned to a ν(CC)_NAP_ mode (CC stretching mode of the naphthalene moieties) on the basis of the normal mode calculation (Table S1 in Supporting Information File 1). Upon second oxidation, the former Raman vibrational band upshifts by 24 cm^−1^ for **1^2+^**, and the naphthalene ν(CC)_NAP_ stretching one disappears. Overall, the evolution of the CBD breathing mode, throughout the series **1→1^•+^→1^2+^** is 1700 **→** 1689 **→** 1713 cm^−1^.

Figure 5 shows the Raman spectra of neutral and oxidized species of **2** (the theoretical Raman spectrum of the model molecule **m-2** (depicted in Figure S1, Supporting Information File 1), which is **2** without the xylyl and methyl substituents can be seen in Figure S3 of Supporting Information File 1). The spectrum of the neutral form consists of a single band at 1704 cm^−1^, assigned to the ν(CC)_exo-CBD_ vibration, which moved to 1689 cm^−1^ in **2^•+^**, and to 1686 cm^−1^ in **2^2+^**. This continuous downshift upon oxidation is in contrast to the observed upshift in **1^•+ ^→ 1^2+^**, corroborating that the fusion topology of the two molecules determines the vibrational dynamics in connection with the fundamental role in the stabilization of the dications.

A second feature, measured at 1596 cm^−1^ in **2**, split into three peaks upon oxidation which are typically arising from ν(CC)_NAP_ modes. However, while the single peak of **1^•+^** at 1567 cm^−1^ can be taken as an indicator of structural uniformity within the NAP rings, the three-folded band in **2^•+^** suggests the presence of different CC bonds in the naphthalene groups. The Raman spectrum of the doubly oxidized form also showed a similar profile to that of **2^•+^**, with the only change of the relative intensities of the 1600–1550 cm^−1^ triplet. This result is fully compatible with the presence of the two rather overlapped (i.e., with similar energies) one-electron oxidations in the cyclic voltammetry of **2**. The spectral resemblances for **2^•+^** and **2^2+^** might also indicate that the positive charge is mainly gathered by the central NAP for **2^•+^**, whereas in **2^2+^** the two charges would be localized towards the outermost NAPs. Nonetheless, **2^•+^** and **2^2+^** both can be viewed as NAPs bearing positive charges.

### Vibrational force field

A suitable tool to visualize the structural impact caused by the topological difference between **1** and **2** is using energetic parameters unequivocally associated to individual bonds. This is the case of the vibrational force constants [22]. They are defined as the second derivative of the molecular energy, in the minimum energy molecular structure, with respect to the nuclear displacement coordinates, which are usually the 3*N* Cartesian coordinates (*N* = number of atoms in the molecule). These Cartesian force constants are meaningless, so that they are transformed to a set of internal vibrational coordinates that account for single molecular motions, as stretchings, bendings or torsions. This procedure has been used here to calculate complete sets of stretching force constants associated to the individual CC bonds of **1** and **2**, hereafter designated as *k*[ν(CC)] (see Supporting Information File 1 for details of these calculations). They account for bond strengths, hence allowing a direct comparison between the bonds of parent molecules. In our case, they will reveal the transformation of the CC bond skeleton upon oxidation.

**Figure 6:**
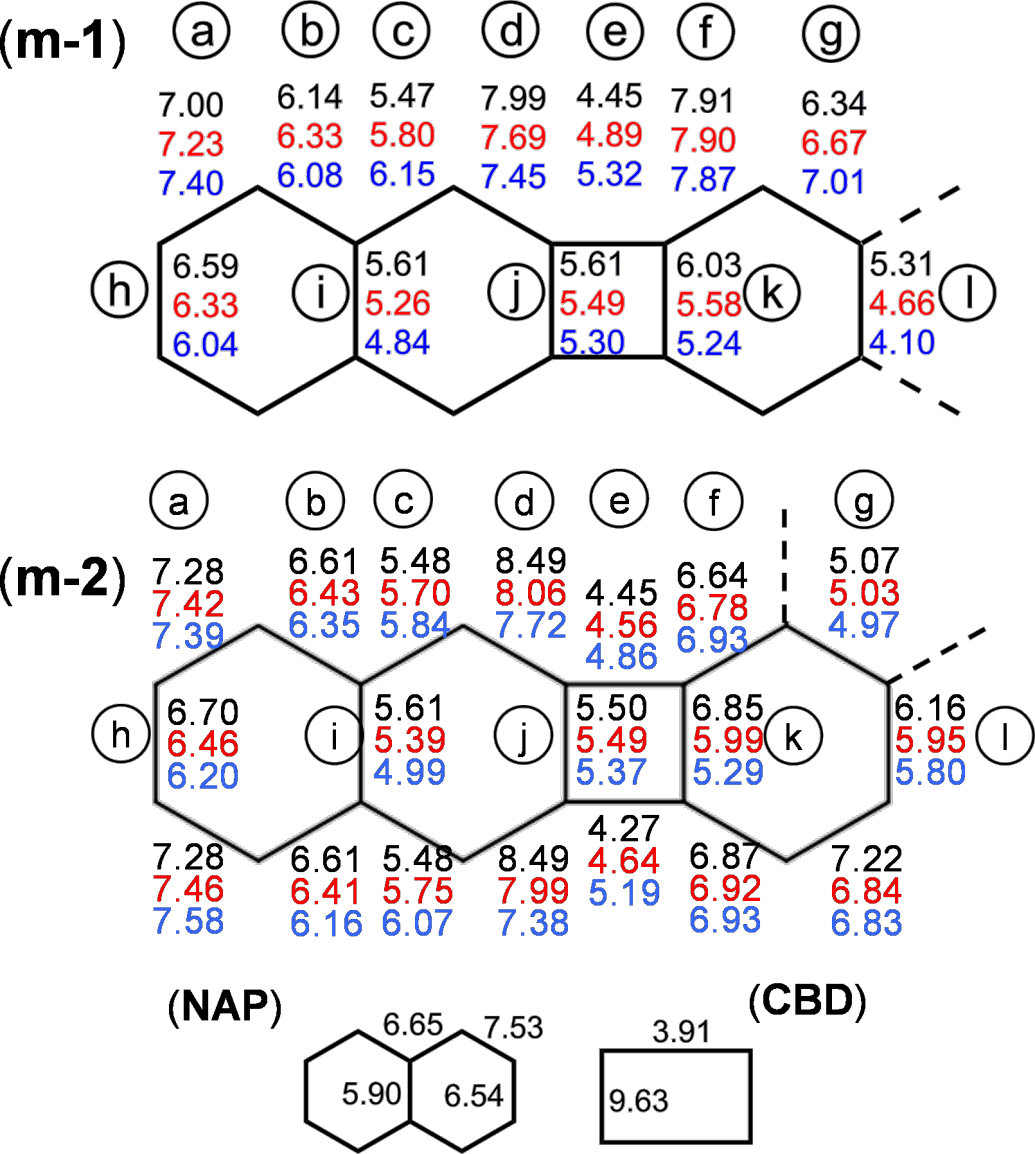
Force constants for the CC stretching vibrational coordinates of the neutral (black), radical cation (red) and dication (blue) of **m-1** and **m-2** compared with those obtained, under the same level or theory, for neutral naphthalene (NAP) and cyclobutadiene (CBD).

Figure 6 summarizes the set of *k*[ν(CC)] values calculated for neutral, radical cation, and dication of **m-1** and **m-2**, as well as those obtained at the same level for individual NAP and CBD. The reliability of the values is supported by the good fit between theoretical and experimental Raman spectra (see Figures S4 and S5 in Supporting Information File 1 for the calculated spectra of the oxidized species). As regarding the neutral species, fusion of these molecular groups provoked an increase of the *k*[ν(CC)] of the four exocyclic CBD bonds (i.e., d bonds in Figure 6) from 7.53 mdyn/Å in pristine NAP to 7.99–7.91 mdyn/Å in **m-1**, which accounts for the high wavenumber value of 1700 cm^−1^ (CBD breathing, or ν(CC)_exo-CBD_) of **1** in comparison with standard CC stretching wavenumbers of isolated NAP (usually lower than 1600 cm^−1^) [23]. Force constants of the j and e CBD bonds in **m-1** are 5.61 and 4.45 mdyn/Å, respectively. Compared with pristine CBD, 9.63 and 3.91 mdyn/Å, this result involves a force constant equalization that clearly reveals the impact of mixing/fusing aromatic and antiaromatic cores. Conversely, the difference of the *k*[ν(CC)] for the adjacent CC bonds of NAP (i.e., d–c bonds), namely 7.99–5.47 mdyn/Å in **m-1** increase compared to 7.53–6.65 mdyn/Å for individual NAP which is compatible with an enhancement of the quinoid character of NAP.

The *k*[ν(CC)] values for exocyclic d bonds of CBD decreased from 7.99 to 7.69 mdyn/Å on going from **m-1** to **m-1^•+^**, which agrees with the observed 1700 → 1689 cm^−1^ downshift of the ν(CC)_exo-CBD_ Raman band. However, the further upshift observed in **m-1^2+^** cannot be justified by an increase of this CC stretching force constant, indeed, it decreases to 7.45 mdyn/Å in the dication. In such cases, the explanation must be sought by analyzing the complete set of CC bond force constants involved in the normal mode of Figure 4, which are those of d, e, f and j bonds. Oxidation decreases the d, f and j force constants while increases the e ones. The *k*[ν(CC)] values of the e bonds (which are the sole CBD bonds non shared with any naphthalene ring) evolves as 4.45 → 4.89 → 5.32 mdyn/Å throughout the series **m-1** → **m-1^•+^** → **m-1^2+^**, which involves differences of 0.44 mdyn/Å for the radical cation and 0.87 for the dication, both with respect to the neutral molecule. For the bonds whose force constants decrease upon oxidation, the greatest deviation is obtained for d (0.30 and 0.54 mdyn/Å, respectively). These values tell us that the second oxidation involves the enhancement on the *k*[ν(CC)] of the intrinsic CBD bond that is significantly higher than the reduction of force constant of the CC bonds shared with the adjacent NAP rings, which justifies the wavenumber upshift from 1689 to 1713 cm^−1^ on the ν(CC)_exo-CBD_ Raman band in the dication.

Despite the symmetry lowering with respect to **m-1**, *D*_2_*_h_* → *C*_2_*_h_*, the force field of neutral **m-2** (Figure 6) seems to preserve the punctual group of its linear analogue, especially concerning the outermost NAP moieties. Interestingly, this *quasi*-*D*_2_*_h_* symmetry is broken upon oxidation, which is supported by the appearance of the triplet of Raman bands in the 1600–1550 cm^−1^ region. On the other hand, the *k*[ν(CC)] values of **m-2** and its cationic species follow all the same qualitative behavior exhibited for **m-1**, though with significant quantitative differences. The force constant of the e bond (intrinsic CBD) in the dication of **m-2** is only 0.41 mdyn/Å higher than in the neutral species, i.e., a reduction of 53% with respect to **m-1**. On the contrary, the decrease for the d bond (NAP adjacent) from **m-2** to **m-2^2+^** is 0.77 mdyn/Å, i.e., 30% higher than in **m-1**. In the case of the j bond, which is shared by CBD and NAP, both trends compensate each other, as its *k*[ν(CC)] decreases, upon double oxidation, 0.13 mdyn/Å for **m-2** and 0.31 mdyn/Å for **m-1**. These data evidence the sensitivity of the ν(CC)_exo-CBD_ Raman band to the fusion topology. In summary, the force constant analysis tells us that the structural changes upon oxidation are largely localized in CBD for **m-1** (marked by the zig-zag shift of the 1700 cm^−1^ Raman band) and in NAP for **m-2** (marked by the triplet in the 1500–1600 cm^−1^ region).

### Nucleus-independent chemical shifts (NICS)

**Figure 7:**
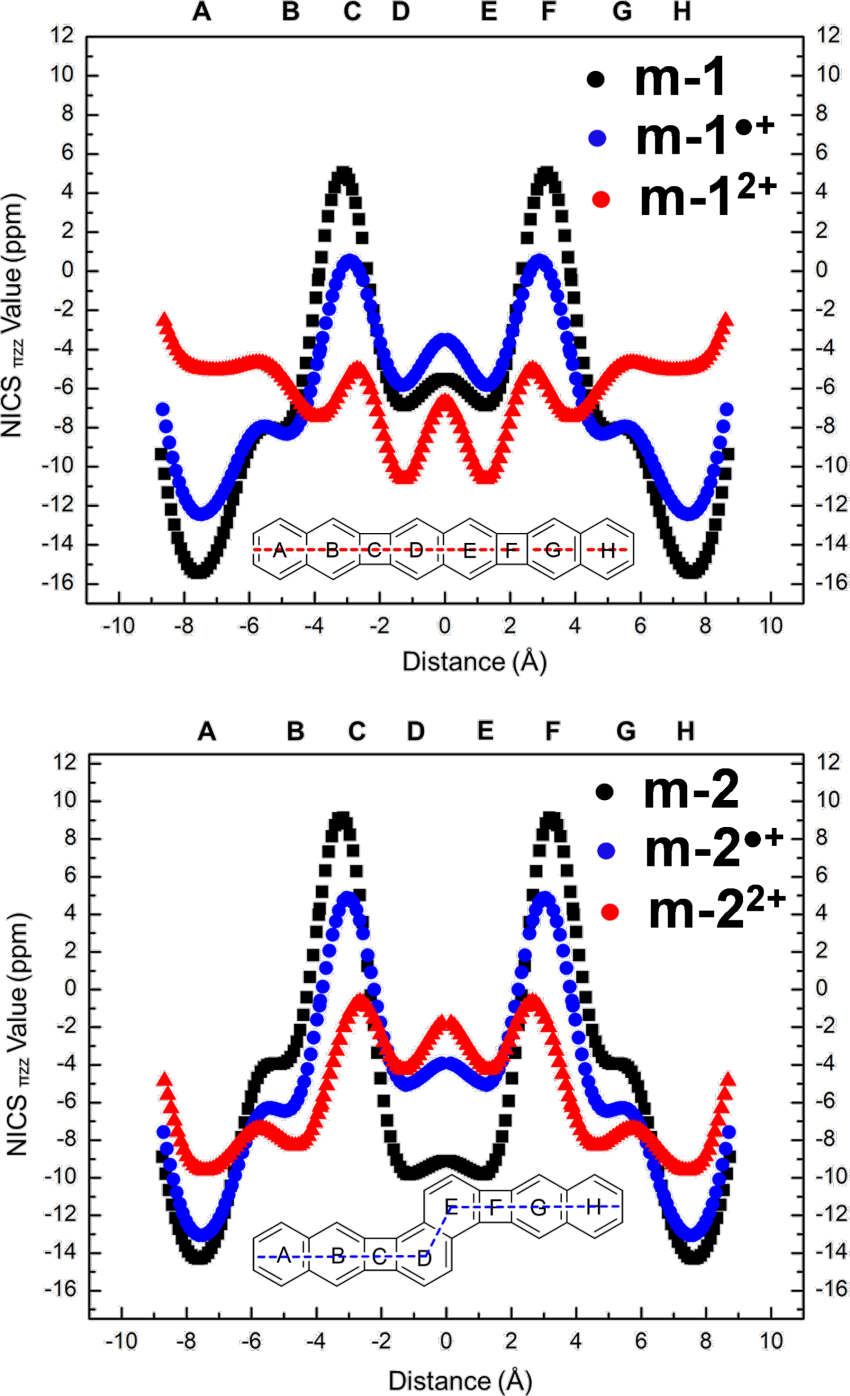
NICS-XY scans, at the (U)B3LYP/6-311G(d,p) level, for neutral **m-1** and **m-2** (black) and for their oxidized radical cations (blue) and dications (red).

Among the different criteria to evaluate aromaticity, magnetic properties are the most confident, as they are directly connected with the ring currents associated to electronic delocalization. In order to figure out the driving force that leads the stabilization of the oxidized species of **1** and **2**, we calculated the NICS values of all fused rings in the neutral and cationic species of **m-1** and **m-2** (Figure 7), in order to obtain a precise and comparable measurement of the aromatic, antiaromatic, or non-aromaticity characters of each ring [24]. They were scanned along the main molecular axis, at a standard distance of 1.7 Å from the molecular plane to discard most of the contribution from the σ orbitals [25–26].

In neutral **m-1**, NAP and CBD rings exhibited negative and positive NICS values, respectively, revealing their aromatic and antiaromatic characters. By oxidizing to **m-1^•+^**, this picture was preserved, though the degrees of aromaticity and antiaromaticity in the constituent rings are reduced with respect to the neutral. Notably, the NICS values of CBD rings in **m-1^•+^** were approaching zero, suggesting these units become non-aromatic. Removing a second electron turns all the rings aromatic, including the CBDs, as indicated by the negative NICS values across the entire molecule. Thus, on **m-1** → **m-1^2+^** transformation, the molecule converges towards a *quasi*-uniform sequence of fused rings, from the aromaticity viewpoint, in which we can stand out the following facts: i) the external/internal NAPs evolve from largely/slightly aromatic to slightly/more aromatic, and ii) the CBDs change from antiaromatic to slightly aromatic. This picture is quantitatively reflected by the difference between the largest (positive) and smallest (negative) NICS values, 8 ppm for **m-1^2+^** versus 23 ppm for **m-1**.

In **2**, the behavior is totally different, since the NICS patterns do not show sign inversion upon oxidation (Figure 7). The **m-2** → **m-2^•+^** transformation localizes the largest changes on the central NAP, which accommodates the positive charges with the two CBD rings and acts as an antiaromatic barrier which prevents outermost charge delocalization. Conversely on **m-2^•+^** → **m-2^2+^**, NICS major changes are concentrated over the external NAP rings, on which the two charges mostly reside. In this case, the CBDs act as stoppers for whole innermost electron delocalization

### Anisotropy of the current induced density (ACID)

**Figure 8:**
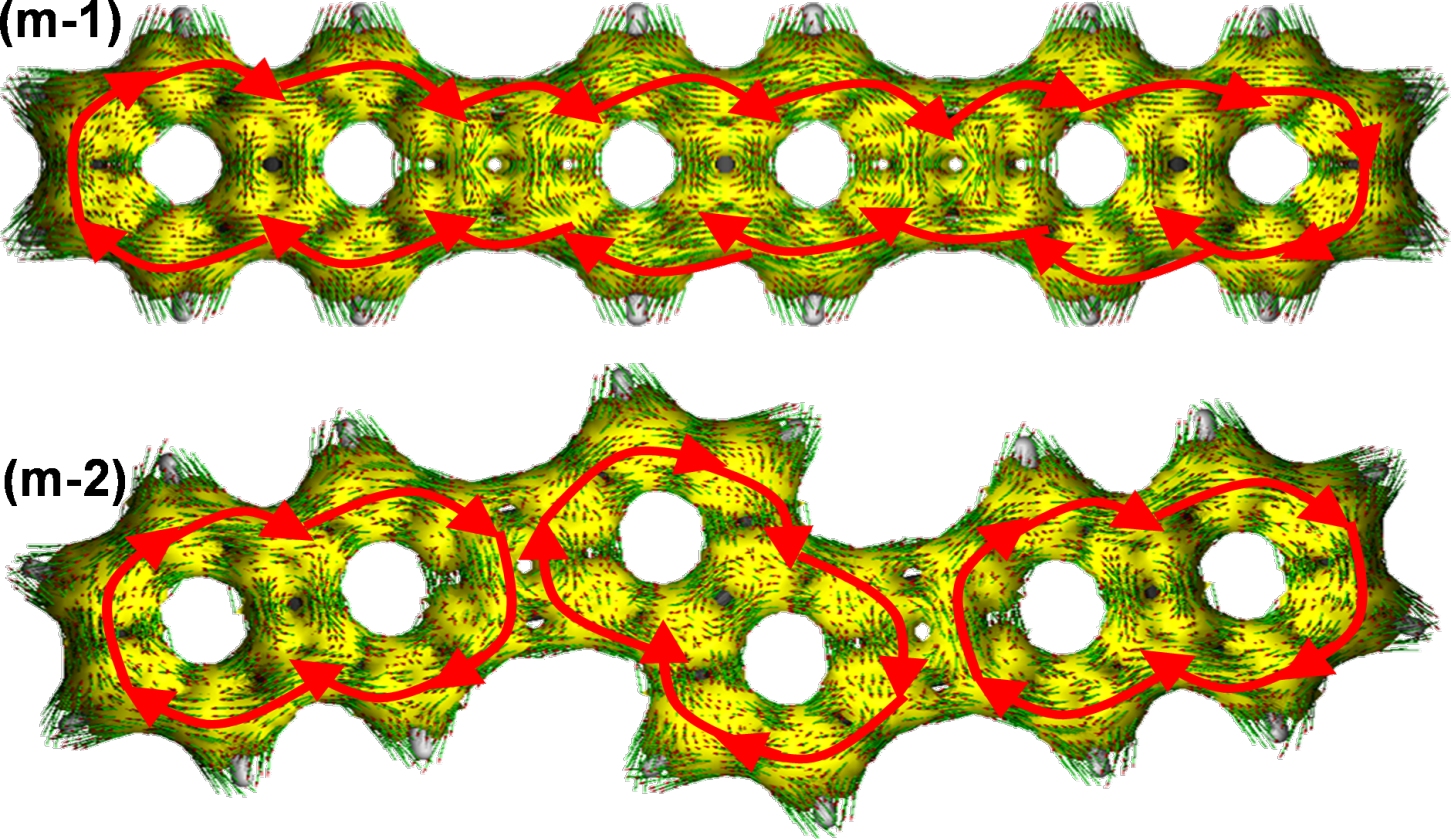
ACID plots at the CSGT-B3LYP/6-311G(d,p) level for dicationic species **m-1^2+^** (top) and **m-2^2+^** (bottom).

The NICS analysis of the precedent paragraph is therefore consistent with the existence of an emergent global diatropic ring current in **m-1^2+^** along the entire molecular perimeter, whereas up to three independent ring currents are expected for the **m-2^2+^** structure. To visualize these results, we have analyzed the ACID plots for the neutral and cationic species of **m-1** and **m-2** [20,26]. Figure 8 shows these plots for the dications, while those of the neutral and radical cation species are included in Figures S6 and S7 of Supporting Information File 1. While the plot of **m-1** contains clockwise and counter-clockwise ring currents in the NAP and CBD units, respectively, **m-1^2+^** clearly showed a global diatropic peripheral ring current. This agrees with the negative NICS values across the entire molecule of **m-1^2+^**. It also justifies the behavior of the Raman bands and the changes in the CC bond force constants. In further agreement with this description, we found that **m-1^2+^** discloses a singlet closed-shell ground electronic state without any trace of diradical character. Thus, the global current [27] provides a unique stabilizing effect for the dication of **m-1**. Such a stabilizing global current was not obvious in **m-2^2+^**. Indeed, local ring currents are more evident in the outermost NAP rings, without showing a net circuit of diatropic current between them. This is consistent with the fact that the ground electronic state of **m-2^2+^** converges into an open-shell diradical structure (more stable than the closed-shell one by 1 kcal/mol at the DFT/(U)B3LYP/6-311G(d,p) level).

Both **1** and **2** are composed of a total of 30 π electrons, which correspond to the Hückel count of 4*n* + 2, with *n* = 7. However, the two systems in their neutral states avoid this formulation and exhibit segmented structures, with attenuated aromatic character of the NAP rings due to the vicinal CBD ones, and mitigated antiaromaticity of the CBD as the result of the vicinal NAP rings. For the dications, the number of π electrons is 4*n* (*n* = 7), it is to say, an antiaromatic Hückel count which is clearly compensated in **m-1^2+^** by the formation of a well-defined diatropic global ring current. In the case of **m-2^2+^**, antiaromaticity of CBD rings still remains because the electronic circulation is split in three independent ring currents.

## Conclusion

Two [3]naphthylene isomers with different fusion topologies, linear (**1**) and angular (**2**), have been studied in their oxidized forms. The first oxidized states of both molecules attend to a concatenation of aromatic NAP and antiaromatic CBD units, in which the stability of the π-electronic structure arises from the dominance of local aromatic segments.

The generation of the linear dication, **1^2+^**, erases this alternation pattern and conversely produces a global diatropic ring current which spreads over the whole molecule along the peripheral ribbons. On the contrary, the angular dication preserves the aromatic–antiaromatic confinement of the neutral and radical cation species. This is an unusual case scarcely described in 1D π-conjugated polycyclic molecules, where the stability of the linear dication is attributed to the formation of a global ring current.

## Experimental

### Electrochemistry

Cyclic voltammetry experiments were conducted with a three-electrode geometry operating with a glassy carbon as the working electrode. A Pt-coil counter electrode. and an Ag wire, as the pseudo-reference, were used. Potential values are given with respect to the ferrocene/ferrocenium (Fc/Fc^+^) couple. Electrolyte solutions, at a concentration 0.1 M, were prepared from anhydrous, degassed HPLC grade CH_2_Cl_2_ and anhydrous Bu_4_NPF_6_. Voltammograms were recorded at a sweep rate of 100 mV s^−1^. Sample concentrations were ca. 1–2 mM.

### Electronic spectroscopy

UV–vis–NIR spectroelectrochemical studies were conducted on a Cary 5000 spectrophotometer. A C3 epsilon potentiostat from BASi was used for the electrolysis using a thin layer cell from a demountable Specac^®^ Omni cell. In this cell, a three-electrode system was coupled to conduct in situ spectroelectrochemistry. A Pt gauze and a Pt wire were used as working and counter electrodes, respectively. A Ag wire was employed as the pseudo-reference electrode in a 0.1 M solution of Bu_4_NPF_6_ in freshly distilled CH_2_Cl_2_. Sample concentration was 1 mM. The spectra were collected by constant potential electrolysis, and the potentials were changed in intervals of 15 mV.

### Raman spectroscopy

Raman spectra were obtained using a Bruker^®^ RAMII Fourier transform Raman spectrometer, purged with dry nitrogen. Excitation radiation at 1064 nm was generated by a Nd-YAG laser working at 500 mW. Backscattering collection of the Raman radiation was performed. Typically, 2000 scans at a resolution better than 4 cm^−1^ were accumulated to optimize the signal-to-noise ratio.

### Theoretical methods

Quantum chemistry was addressed with the Gaussian 09 suite of programs [28]. DFT calculations were performed at the (U)B3LYP[29–30] level, using the 6-311G* basis set [31]. This includes polarization functions on heavy atoms, being necessary for calculations on charged species. Geometry optimizations were achieved by allowing all the geometrical parameters to vary independently. The optimum energy structures were found to be a true minimum in the ground state potential energy surface. Analytical harmonic force constants, in Cartesian coordinates, and Raman intensities were evaluated at the ground-state-optimized geometry. The theoretical spectra were obtained by convoluting the calculated frequencies with Lorentzian functions. Bond stretching force constants were obtained using the given molecular symmetry for in-plane vibrations (see Supporting Information File 1).

Nucleus independent chemical shifts (NICS) were computed at the GIAO-B3LYP/6-311G* level. Calculations were carried out using the Aroma package [32], accordingly to published procedures [33]. The ACID plots were generated using the continuous set of gauge transformations (CSGT) method, as implemented in the Gaussian 09 suite, and the AICD 2.0.0 program [34].

## Supporting Information

File 1 Chemical structures of **m-1** and **m-2**, vibrational assignment of **m-1**, the 1730 cm^−1^ normal mode, theoretical Raman spectrum of **m-1**, details of the force field calculations, theoretical Raman spectra of **m-1** and **m-2**, and ACID plots of **m-1** and **m-2**.

## Data Availability

Additional research data generated and analyzed during this study is not shared.

## References

[1] Brédas J L, Street G B (1985). Acc Chem Res.

[2] Perepichka I F, Perepichka D F (2009). Handbook of Thiophene-Based Materials: Applications in Organic Electronics and Photonics.

[3] Furukawa Y (1996). J Phys Chem.

[4] Tönshoff C, Bettinger H F (2021). Chem – Eur J.

[5] Einholz R, Bettinger H F (2013). Angew Chem, Int Ed.

[6] Plasser F, Pašalić H, Gerzabek M H, Libisch F, Reiter R, Burgdörfer J, Müller T, Shepard R, Lischka H (2013). Angew Chem.

[7] Zade S S, Zamoshchik N, Reddy A R, Fridman-Marueli G, Sheberla D, Bendikov M (2011). J Am Chem Soc.

[8] Breslow R (1973). Acc Chem Res.

[9] Fujii S, Marqués-González S, Shin J-Y, Shinokubo H, Masuda T, Nishino T, Arasu N P, Vázquez H, Kiguchi M (2017). Nat Commun.

[10] Breslow R, Murayama D R, Murahashi S-I, Grubbs R (1973). J Am Chem Soc.

[11] Chen W, Li H, Widawsky J R, Appayee C, Venkataraman L, Breslow R (2014). J Am Chem Soc.

[12] Breslow R, Foss F W (2008). J Phys: Condens Matter.

[13] Rudebusch G E, Espejo G L, Zafra J L, Peña-Alvarez M, Spisak S N, Fukuda K, Wei Z, Nakano M, Petrukhina M A, Casado J (2016). J Am Chem Soc.

[14] Barker J E, Dressler J J, Cárdenas Valdivia A, Kishi R, Strand E T, Zakharov L N, MacMillan S N, Gómez-García C J, Nakano M, Casado J (2020). J Am Chem Soc.

[15] Peeks M D, Jirasek M, Claridge T D W, Anderson H L (2019). Angew Chem, Int Ed.

[16] Jirásek M, Anderson H L, Peeks M D (2021). Acc Chem Res.

[17] Ren L, Gopalakrishna T Y, Park I-H, Han Y, Wu J (2020). Angew Chem, Int Ed.

[18] Jin Z, Teo Y C, Teat S J, Xia Y (2017). J Am Chem Soc.

[19] Jin Z, Yao Z-F, Barker K P, Pei J, Xia Y (2019). Angew Chem, Int Ed.

[20] Jin Z, Teo Y C, Zulaybar N G, Smith M D, Xia Y (2017). J Am Chem Soc.

[21] Müllen K, Wegner G (1998). Electronic Materials: The Oligomer Approach.

[22] Wilson E B, Decius J C, Cross P C (1955). Phys Today.

[23] Martin J M L, El-Yazal J, François J-P (1996). J Phys Chem.

[24] Chen Z, Wannere C S, Corminboeuf C, Puchta R, Schleyer P v R (2005). Chem Rev.

[25] Stanger A (2006). J Org Chem.

[26] Gershoni‐Poranne R, Stanger A (2014). Chem – Eur J.

[27] Herges R, Geuenich D (2001). J Phys Chem A.

[28] (2010). Gaussian 09.

[29] Becke A D (1993). J Chem Phys.

[30] Lee C, Yang W, Parr R G (1988). Phys Rev B.

[31] Clark T, Chandrasekhar J, Spitznagel G W, Schleyer P V R (1983). J Comput Chem.

[R32] 32AROMA plugin; Rahalkar, A.; Stanger, A. This software may be downloaded free of charge from https://chemistry.technion.ac.il/en/team/amnon-stanger/

[33] Gershoni-Poranne R, Stanger A (2015). Chem Soc Rev.

[34] Geuenich D, Hess K, Köhler F, Herges R (2005). Chem Rev.

