# Supplementary material for: Oxidation of [3]naphthylenes to cations and dications converts local paratropicity into global diatropicity
Source: Beilstein J Org Chem. 2025 Feb 5;21:277–85. doi: 10.3762/bjoc.21.20 (PMC11809581; doi:10.3762/bjoc.21.20)

BEILSTEIN JOURNAL OF ORGANIC CHEMISTRY

Supporting Information

for

Oxidation of [3]naphthylenes to cations and dications converts local paratropicity into global diatropicity

Abel Cárdenas, Zexin Jin, Yong Ni, Jishan Wu, Yan Xia, Francisco Javier Ramírez and Juan Casado

Beilstein J. Org. Chem. **2025,** *21,* 277–285. doi:10.3762/bjoc.21.20

Chemical structures of m-1 and m-2, vibrational assignment of m-1, the 1730 cm^{-1} normal mode, theoretical Raman spectrum of m-1, details of the force field calculations, theoretical Raman spectra of m-1 and m-2, and ACID plots of m-1 and m-2

License and Terms: This is a supporting information file under the terms of the Creative Commons Attribution License (https://creativecommons.org/licenses/by/4.0). Please note that the reuse, redistribution and reproduction in particular requires that the author(s) and source are credited and that individual graphics may be subject to special legal provisions.
The license is subject to the *Beilstein Journal of Organic Chemistry* terms and conditions: (https://www.beilstein-journals.org/bjoc/terms)

Table of contents

Chemical structures of **m-1** and **m-2** S2

Basic quantum chemical data S3

Vibrational assignment of **m-1** S23

The 1730 cm^{-1} normal mode S23

Theoretical Raman spectrum of **m-2** S24

Details of force field calculations S24

Theoretical Raman spectra of **m-1** and **m-2** S26

ACID plots of **m-1** and **m-2** S27

Figure S1. Chemical structures of the model compounds **m-1** and **m-2.**

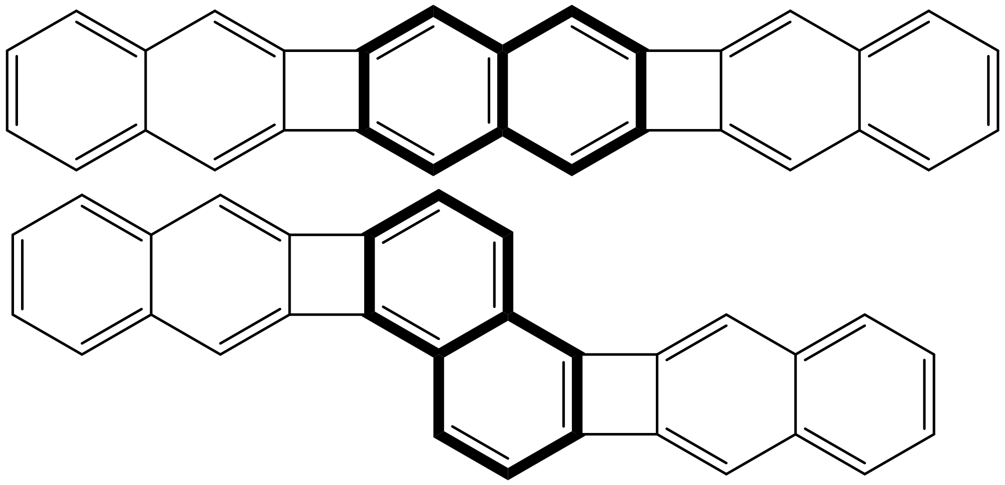

Basic quantum chemical data.
Optimized geometries.

Table S1. Z-matrix of the B3LYP/6-311G(d,p)-optimized geometry of **m-1**.

0 1

C	-0.70408500	0.11672000	8.73125500
C	0.70408500	-0.11672000	8.73125500
C	1.34909700	-0.25463200	7.50359500
C	0.70539200	-0.14418200	6.25455500
C	-0.70539200	0.14418200	6.25455500
C	-1.34909700	0.25463200	7.50359500
C	1.45068600	-0.30477300	5.01506000
C	0.70728800	-0.14975700	3.88673600
C	-0.70728800	0.14975700	3.88673600
C	-1.45068600	0.30477300	5.01506000
C	0.70369600	-0.14924700	2.39321300
C	-0.70369600	0.14924700	2.39321300
C	1.41524700	-0.30021000	1.23862600
C	0.70859700	-0.15030800	0.00000000
C	-0.70859700	0.15030800	0.00000000
C	-1.41524700	0.30021000	1.23862600
C	1.41524700	-0.30021000	-1.23862600
C	0.70369600	-0.14924700	-2.39321300
C	-0.70369600	0.14924700	-2.39321300
C	-1.41524700	0.30021000	-1.23862600
C	0.70728800	-0.14975700	-3.88673600
C	-0.70728800	0.14975700	-3.88673600
C	1.45068600	-0.30477300	-5.01506000
C	0.70539200	-0.14418200	-6.25455500
C	-0.70539200	0.14418200	-6.25455500
C	-1.45068600	0.30477300	-5.01506000
C	1.34909700	-0.25463200	-7.50359500
C	0.70408500	-0.11672000	-8.73125500
C	-0.70408500	0.11672000	-8.73125500
C	-1.34909700	0.25463200	-7.50359500
H	2.41497100	-0.45602500	7.51926900
H	-2.41497100	0.45602500	7.51926900
H	2.47779500	-0.52559200	1.21640200
H	-2.47779500	0.52559200	1.21640200
H	2.47779500	-0.52559200	-1.21640200
H	-2.47779500	0.52559200	-1.21640200
H	2.41497100	-0.45602500	-7.51926900
H	-2.41497100	0.45602500	-7.51926900
C	1.55806400	-0.18288100	9.96896700
C	2.48022200	0.86475500	10.22014900
C	1.51853100	-1.29715700	10.83779500
C	3.28432400	0.81033300	11.36503100
C	2.34807100	-1.31797500	11.96573600
C	3.21665700	-0.26792100	12.24160400
H	3.97700200	1.62477300	11.56112500
H	2.31202100	-2.17913600	12.62794400

H	3.84851900	-0.29657200	13.12478100
C	-1.55806400	0.18288100	9.96896700
C	-2.48022200	-0.86475500	10.22014900
C	-1.51853100	1.29715700	10.83779500
C	-3.28432400	-0.81033300	11.36503100
C	-2.34807100	1.31797500	11.96573600
C	-3.21665700	0.26792100	12.24160400
H	-3.97700200	-1.62477300	11.56112500
H	-2.31202100	2.17913600	12.62794400
H	-3.84851900	0.29657200	13.12478100
C	1.55806400	-0.18288100	-9.96896700
C	1.51853100	-1.29715700	-10.83779500
C	2.48022200	0.86475500	-10.22014900
C	2.34807100	-1.31797500	-11.96573600
C	3.28432400	0.81033300	-11.36503100
C	3.21665700	-0.26792100	-12.24160400
H	2.31202100	-2.17913600	-12.62794400
H	3.97700200	1.62477300	-11.56112500
H	3.84851900	-0.29657200	-13.12478100
C	-1.55806400	0.18288100	-9.96896700
C	-1.51853100	1.29715700	-10.83779500
C	-2.48022200	-0.86475500	-10.22014900
C	-2.34807100	1.31797500	-11.96573600
C	-3.28432400	-0.81033300	-11.36503100
C	-3.21665700	0.26792100	-12.24160400
H	-2.31202100	2.17913600	-12.62794400
H	-3.97700200	-1.62477300	-11.56112500
H	-3.84851900	0.29657200	-13.12478100
C	2.66069300	2.03698900	9.27674500
H	3.34499800	1.78540700	8.45754900
H	1.72441100	2.35619100	8.81666100
H	3.09273600	2.89109900	9.80631600
C	0.63355500	-2.49229800	10.57453700
H	-0.37965500	-2.33078900	10.95544100
H	0.55309900	-2.70770100	9.50578200
H	1.03420100	-3.38142800	11.06978300
C	-0.63355500	2.49229800	10.57453700
H	0.37965500	2.33078900	10.95544100
H	-0.55309900	2.70770100	9.50578200
H	-1.03420100	3.38142800	11.06978300
C	-2.66069300	-2.03698900	9.27674500
H	-3.09273600	-2.89109900	9.80631600
H	-3.34499800	-1.78540700	8.45754900
H	-1.72441100	-2.35619100	8.81666100
C	-0.63355500	2.49229800	-10.57453700
H	-0.55309900	2.70770100	-9.50578200
H	0.37965500	2.33078900	-10.95544100
H	-1.03420100	3.38142800	-11.06978300
C	-2.66069300	-2.03698900	-9.27674500
H	-1.72441100	-2.35619100	-8.81666100
H	-3.34499800	-1.78540700	-8.45754900
H	-3.09273600	-2.89109900	-9.80631600

C	0.63355500	-2.49229800	-10.57453700
H	0.55309900	-2.70770100	-9.50578200
H	-0.37965500	-2.33078900	-10.95544100
H	1.03420100	-3.38142800	-11.06978300
C	2.66069300	2.03698900	-9.27674500
H	1.72441100	2.35619100	-8.81666100
H	3.34499800	1.78540700	-8.45754900
H	3.09273600	2.89109900	-9.80631600
C	-2.92369100	0.61518800	-4.99770400
H	-3.14624700	1.55507300	-5.51621600
H	-3.51009900	-0.16986300	-5.48971000
H	-3.28477400	0.70633300	-3.97072900
C	2.92369100	-0.61518800	-4.99770400
H	3.14624700	-1.55507300	-5.51621600
H	3.51009900	0.16986300	-5.48971000
H	3.28477400	-0.70633300	-3.97072900
C	-2.92369100	0.61518800	4.99770400
H	-3.51009900	-0.16986300	5.48971000
H	-3.14624700	1.55507300	5.51621600
H	-3.28477400	0.70633300	3.97072900
C	2.92369100	-0.61518800	4.99770400
H	3.51009900	0.16986300	5.48971000
H	3.14624700	-1.55507300	5.51621600
H	3.28477400	-0.70633300	3.97072900

Table S2. Z-matrix of the UB3LYP/6-311G(d,p)-optimized geometry of the radical cation of **m-1**.

1 2

C	8.69184500	0.71024800	-0.11687600
C	8.69184700	-0.71022700	0.11689400
C	7.46908200	-1.35519400	0.25321900
C	6.21657200	-0.71181900	0.14330900
C	6.21657100	0.71183400	-0.14328000
C	7.46907900	1.35521000	-0.25320200
C	4.99147900	-1.46066500	0.30640800
C	3.85828300	-0.71112100	0.15104100
C	3.85828200	0.71113200	-0.15100800
C	4.99147700	1.46067900	-0.30637900
C	2.38556800	-0.71209000	0.15131000
C	2.38556600	0.71210000	-0.15127100
C	1.23099500	-1.42788300	0.30309000
C	0.00000000	-0.72160800	0.15315600
C	-0.00000200	0.72161600	-0.15310700
C	1.23099200	1.42789200	-0.30304600
C	-1.23099400	-1.42788400	0.30309300
C	-2.38556900	-0.71209200	0.15131500
C	-2.38557000	0.71209800	-0.15126100
C	-1.23099700	1.42789200	-0.30304200
C	-3.85828500	-0.71112600	0.15104400
C	-3.85828600	0.71112900	-0.15099500
C	-4.99148200	-1.46067500	0.30640000
C	-6.21657500	-0.71183100	0.14329600

C	-6.21657600	0.71182500	-0.14327500
C	-4.99148300	1.46067400	-0.30636200
C	-7.46908600	-1.35520700	0.25321100
C	-8.69185100	-0.71024600	0.11688300
C	-8.69185300	0.71022600	-0.11690300
C	-7.46908800	1.35519700	-0.25320200
H	7.48600200	-2.41948600	0.45776100
H	7.48599700	2.41949900	-0.45776100
H	1.21053600	-2.48921000	0.52842700
H	1.21053100	2.48921900	-0.52838200
H	-1.21053400	-2.48921200	0.52842600
H	-1.21053800	2.48921900	-0.52837500
H	-7.48600300	-2.41949600	0.45777100
H	-7.48600800	2.41948800	-0.45774800
C	9.93100200	-1.55522300	0.19319600
C	10.17412300	-2.50324200	-0.83415300
C	10.80711600	-1.47772300	1.30073600
C	11.32515900	-3.29609900	-0.76795800
C	11.93769700	-2.30216600	1.33268800
C	12.20920600	-3.19482900	0.30190200
H	11.52173500	-4.00636100	-1.56632100
H	12.60566300	-2.24336300	2.18751000
H	13.09590700	-3.82040800	0.33924500
C	9.93100600	1.55523300	-0.19321600
C	10.17418800	2.50323400	0.83413800
C	10.80708500	1.47772100	-1.30078300
C	11.32523900	3.29606700	0.76790700
C	11.93768000	2.30214200	-1.33277000
C	12.20924100	3.19479400	-0.30198800
H	11.52186200	4.00631100	1.56627500
H	12.60561500	2.24333000	-2.18761600
H	13.09595600	3.82035200	-0.33935500
C	-9.93101400	-1.55521900	0.19322200
C	-10.80709600	-1.47769000	1.30078200
C	-10.17418000	-2.50323700	-0.83411400
C	-11.93767800	-2.30213000	1.33278700
C	-11.32521400	-3.29609300	-0.76786800
C	-12.20922400	-3.19480800	0.30202200
H	-12.60561300	-2.24331500	2.18763300
H	-11.52182200	-4.00635900	-1.56622000
H	-13.09593200	-3.82037700	0.33939900
C	-9.93101100	1.55520900	-0.19324300
C	-10.80707600	1.47771000	-1.30082000
C	-10.17417500	2.50321600	0.83410200
C	-11.93764800	2.30216500	-1.33282400
C	-11.32520000	3.29608600	0.76785500
C	-12.20919900	3.19482600	-0.30204600
H	-12.60557400	2.24337100	-2.18767800
H	-11.52180700	4.00634400	1.56621300
H	-13.09589600	3.82040900	-0.33942700
C	9.22943700	-2.71674300	-2.00026300
H	8.42521400	-3.41535400	-1.73887500

H	8.75359400	-1.79450300	-2.33791800
H	9.76476700	-3.15152900	-2.84852700
C	10.54915300	-0.56942100	2.47987500
H	10.96102000	0.43044800	2.31232700
H	9.48115100	-0.45857200	2.68559700
H	11.02159600	-0.97184500	3.37991200
C	10.54907000	0.56942100	-2.47991200
H	10.96096800	-0.43044100	-2.31239500
H	9.48105900	0.45855300	-2.68557300
H	11.02145400	0.97185900	-3.37997500
C	9.22957800	2.71672200	2.00031100
H	9.76497700	3.15143400	2.84856900
H	8.42538200	3.41539500	1.73900200
H	8.75369900	1.79449300	2.33794500
C	-10.54905800	0.56940400	-2.47994600
H	-9.48104600	0.45854900	-2.68561800
H	-10.96093500	-0.43046400	-2.31241600
H	-11.02145800	0.97182800	-3.38000500
C	-9.22953200	2.71669100	2.00025000
H	-8.75380000	1.79442000	2.33798500
H	-8.42522200	3.41520300	1.73886500
H	-9.76486400	3.15158000	2.84845900
C	-10.54908400	-0.56936500	2.47989400
H	-9.48107200	-0.45846700	2.68553800
H	-10.96100800	0.43048500	2.31236800
H	-11.02144500	-0.97180000	3.37997000
C	-9.22954600	-2.71673200	-2.00026700
H	-8.75372100	-1.79449000	-2.33794700
H	-8.42530900	-3.41534000	-1.73890800
H	-9.76490900	-3.15152400	-2.84850700
C	-4.97081900	2.93235900	-0.61617200
H	-5.49116300	3.15215500	-1.55465000
H	-5.46097900	3.51563400	0.17101900
H	-3.94477000	3.29397700	-0.70964200
C	-4.97081500	-2.93235800	0.61621600
H	-5.49117400	-3.15215300	1.55468700
H	-5.46095800	-3.51563900	-0.17098100
H	-3.94476600	-3.29397100	0.70970600
C	4.97081000	2.93236100	-0.61620100
H	5.46095700	3.51564500	0.17099100
H	5.49116600	3.15215000	-1.55467400
H	3.94476100	3.29397400	-0.70968800
C	4.97081600	-2.93234900	0.61622400
H	5.46096700	-3.51562900	-0.17096800
H	5.49117100	-3.15214000	1.55469800
H	3.94476800	-3.29396500	0.70970800

Table S3. Z-matrix of the B3LYP/6-311G(d,p)-optimized geometry of the closed-shell dication of **m-1**.

A) B3LYP/6-311G(D,P) CLOSED-SHELL STATE OF THE DICATION OF m-1.

2 1

C	8.67091600	0.71606400	-0.11733700
C	8.67092200	-0.71590200	0.11753800
C	7.44797700	-1.36597200	0.22647000
C	6.19758900	-0.72109800	0.12540500
C	6.19758200	0.72121500	-0.12527800
C	7.44796100	1.36610300	-0.22634500
C	4.97805400	-1.47386800	0.28155100
C	3.84428700	-0.71776300	0.14063900
C	3.84427900	0.71783000	-0.14062100
C	4.97803700	1.47395700	-0.28148900
C	2.38248800	-0.71785100	0.13967800
C	2.38248300	0.71789400	-0.13970700
C	1.22698900	-1.43907000	0.27839100
C	-0.00005100	-0.73080800	0.14110600
C	-0.00005500	0.73082400	-0.14118200
C	1.22697700	1.43910000	-0.27844200
C	-1.22708300	-1.43907900	0.27838600
C	-2.38258900	-0.71787400	0.13964500
C	-2.38259300	0.71786600	-0.13977600
C	-1.22709400	1.43908400	-0.27848700
C	-3.84438200	-0.71780200	0.14059200
C	-3.84439000	0.71778100	-0.14072300
C	-4.97814200	-1.47391400	0.28154100
C	-6.19768200	-0.72116500	0.12535000
C	-6.19768900	0.72112900	-0.12542400
C	-4.97815500	1.47388700	-0.28164100
C	-7.44806600	-1.36602700	0.22649500
C	-8.67103100	-0.71599100	0.11748900
C	-8.67103800	0.71595000	-0.11750500
C	-7.44807700	1.36598800	-0.22653400
H	7.46272400	-2.42904400	0.43261500
H	7.46268700	2.42915000	-0.43260600
H	1.20807300	-2.50385100	0.48483600
H	1.20805500	2.50388200	-0.48488800
H	-1.20816100	-2.50385800	0.48484300
H	-1.20817600	2.50386400	-0.48494100
H	-7.46280100	-2.42907000	0.43278200
H	-7.46282000	2.42903300	-0.43281000
C	9.91444800	-1.52691000	0.24732700
C	10.15110500	-2.58665900	-0.67622000
C	10.82996000	-1.29844100	1.31291800
C	11.33297100	-3.32020600	-0.56671400
C	11.98876900	-2.07658500	1.38976900
C	12.25374800	-3.06764500	0.45222900
H	11.53702400	-4.10222500	-1.29210400
H	12.68027200	-1.90959300	2.21041600
H	13.16277600	-3.65678900	0.52149100

C	9.91446800	1.52700400	-0.24730300
C	10.15133500	2.58681700	0.67614600
C	10.82993300	1.29826000	-1.31286800
C	11.33331100	3.32015600	0.56651400
C	11.98887500	2.07620300	-1.38983700
C	12.25402400	3.06733500	-0.45243500
H	11.53753100	4.10220200	1.29182600
H	12.68034200	1.90898200	-2.21046800
H	13.16314800	3.65631900	-0.52177600
C	-9.91450300	-1.52706800	0.24752400
C	-10.82984900	-1.29857100	1.31324500
C	-10.15122700	-2.58695700	-0.67587000
C	-11.98852800	-2.07687300	1.39042700
C	-11.33297200	-3.32065200	-0.56603100
C	-12.25356100	-3.06809800	0.45307300
H	-12.67989300	-1.90987000	2.21118700
H	-11.53707600	-4.10277300	-1.29129700
H	-13.16249300	-3.65735900	0.52260300
C	-9.91449800	1.52705100	-0.24749900
C	-10.82989400	1.29860600	-1.31318900
C	-10.15113500	2.58694400	0.67590600
C	-11.98852400	2.07697900	-1.39032900
C	-11.33286300	3.32068100	0.56614100
C	-12.25349200	3.06819200	-0.45293900
H	-12.67992100	1.91002900	-2.21107300
H	-11.53690400	4.10279600	1.29143000
H	-13.16240200	3.65749000	-0.52243100
C	9.19811400	-2.95180500	-1.79659600
H	8.45815000	-3.69021400	-1.46488800
H	8.64732500	-2.09617000	-2.19072800
H	9.74662100	-3.40779200	-2.62440000
C	10.57063500	-0.31627500	2.43179900
H	11.15732500	0.59865300	2.30501400
H	9.51908200	-0.03694300	2.51145300
H	10.86526600	-0.75851300	3.38790400
C	10.57044500	0.31600800	-2.43163400
H	11.15758200	-0.59867600	-2.30515000
H	9.51897500	0.03624700	-2.51079000
H	10.86443000	0.75839100	-3.38787600
C	9.19852700	2.95213700	1.79662200
H	9.74711900	3.40867500	2.62406500
H	8.45819300	3.69013300	1.46483500
H	8.64817100	2.09646700	2.19128900
C	-10.57064000	0.31611500	-2.43179700
H	-9.51892200	0.03752300	-2.51201300
H	-11.15654200	-0.59919700	-2.30416200
H	-10.86625400	0.75766200	-3.38790900
C	-9.19829900	2.95201400	1.79644300
H	-8.64800900	2.09624400	2.19097800
H	-8.45791200	3.69000700	1.46476500
H	-9.74685700	3.40846900	2.62395500
C	-10.57048400	-0.31614500	2.43188600

H	-9.51878900	-0.03742600	2.51191500
H	-11.15653400	0.59910300	2.30446600
H	-10.86583400	-0.75783100	3.38801800
C	-9.19849100	-2.95206300	-1.79648300
H	-8.64802700	-2.09635400	-2.19089500
H	-8.45826800	-3.69027800	-1.46492700
H	-9.74716600	-3.40827300	-2.62405400
C	-4.95621900	2.95042900	-0.56381000
H	-5.47449300	3.18678200	-1.49919600
H	-5.44556500	3.51843200	0.23449300
H	-3.93053700	3.31367900	-0.65009700
C	-4.95618800	-2.95045400	0.56371500
H	-5.47442400	-3.18680600	1.49912300
H	-5.44556500	-3.51846100	-0.23456600
H	-3.93050100	-3.31370000	0.64996000
C	4.95608100	2.95051100	-0.56359600
H	5.44536100	3.51849100	0.23476400
H	5.47440500	3.18692200	-1.49894000
H	3.93039400	3.31373700	-0.64993000
C	4.95611800	-2.95042800	0.56363100
H	5.44545500	-3.51838000	-0.23471500
H	5.47439900	-3.18684300	1.49899800
H	3.93043600	-3.31368100	0.64990300

B) UB3LYP/6-311G(D,P) OPEN-SHELL BROKEN SYMMETRY - DICATION OF M-1.

2 1

C	8.67100500	0.71598000	-0.11755300
C	8.67100200	-0.71597500	0.11741700
C	7.44805000	-1.36602600	0.22638700
C	6.19767200	-0.72114100	0.12530200
C	6.19767500	0.72116800	-0.12535800
C	7.44805500	1.36604100	-0.22648100
C	4.97812300	-1.47389500	0.28147100
C	3.84436500	-0.71778000	0.14062300
C	3.84436600	0.71783800	-0.14054700
C	4.97812800	1.47393700	-0.28146400
C	2.38256400	-0.71784600	0.13971300
C	2.38256600	0.71791400	-0.13960300
C	1.22706200	-1.43906700	0.27834600
C	0.00002700	-0.73078300	0.14112900
C	0.00003000	0.73085800	-0.14102200
C	1.22706400	1.43913700	-0.27824500
C	-1.22700800	-1.43906300	0.27833200
C	-2.38250900	-0.71783500	0.13970700
C	-2.38250700	0.71792900	-0.13957700
C	-1.22700500	1.43914700	-0.27822200
C	-3.84430700	-0.71776800	0.14061900
C	-3.84431200	0.71785900	-0.14050100
C	-4.97806500	-1.47389300	0.28143200
C	-6.19761500	-0.72113800	0.12530300

C	-6.19762300	0.72118800	-0.12527400
C	-4.97807200	1.47396200	-0.28136700
C	-7.44799100	-1.36604500	0.22632800
C	-8.67093500	-0.71598200	0.11739600
C	-8.67094100	0.71599100	-0.11743600
C	-7.44800000	1.36607300	-0.22633700
H	7.46278700	-2.42908600	0.43258900
H	7.46279800	2.42910600	-0.43266000
H	1.20813800	-2.50386800	0.48469700
H	1.20814400	2.50393800	-0.48460100
H	-1.20809000	-2.50386600	0.48467700
H	-1.20808100	2.50394900	-0.48457000
H	-7.46272800	-2.42912400	0.43243400
H	-7.46275100	2.42915300	-0.43244100
C	9.91452400	-1.52699800	0.24747900
C	10.15144200	-2.58678000	-0.67598400
C	10.82969800	-1.29854100	1.31335600
C	11.33327100	-3.32032600	-0.56612700
C	11.98844100	-2.07674800	1.39060000
C	12.25371200	-3.06779000	0.45312400
H	11.53754700	-4.10235200	-1.29144600
H	12.67967700	-1.90979000	2.21147600
H	13.16270300	-3.65695600	0.52267300
C	9.91453500	1.52698700	-0.24756900
C	10.15140500	2.58677800	0.67589200
C	10.82981400	1.29847200	-1.31334600
C	11.33326400	3.32029000	0.56612300
C	11.98861800	2.07660300	-1.39047200
C	12.25381600	3.06767500	-0.45301200
H	11.53749400	4.10233000	1.29144000
H	12.67994700	1.90956700	-2.21125400
H	13.16284300	3.65679700	-0.52247100
C	-9.91449800	-1.52696900	0.24731000
C	-10.82972300	-1.29856100	1.31315100
C	-10.15147500	-2.58657400	-0.67633600
C	-11.98858400	-2.07662100	1.39018400
C	-11.33339400	-3.32000200	-0.56666400
C	-12.25388900	-3.06750100	0.45255500
H	-12.67987600	-1.90967500	2.21101600
H	-11.53771600	-4.10189300	-1.29211600
H	-13.16295800	-3.65656600	0.52194000
C	-9.91452200	1.52693400	-0.24734700
C	-10.82981100	1.29840900	-1.31311300
C	-10.15147300	2.58662000	0.67621700
C	-11.98872400	2.07639400	-1.39011800
C	-11.33342700	3.31998900	0.56656100
C	-12.25400100	3.06734300	-0.45255500
H	-12.68006600	1.90934800	-2.21088800
H	-11.53772600	4.10194500	1.29194900
H	-13.16310200	3.65636200	-0.52191800
C	9.19878600	-2.95192700	-1.79664300
H	8.45845600	-3.69001900	-1.46506100

H	8.64844500	-2.09621000	-2.19121000
H	9.74748400	-3.40830000	-2.62410900
C	10.57006700	-0.31628000	2.43207600
H	11.15570200	0.59923000	2.30461200
H	9.51825800	-0.03801900	2.51225500
H	10.86574300	-0.75786300	3.38815200
C	10.57023000	0.31625400	-2.43211500
H	11.15634600	-0.59899900	-2.30501000
H	9.51852800	0.03752600	-2.51197400
H	10.86536000	0.75813000	-3.38823000
C	9.19868300	2.95193000	1.79649500
H	9.74734300	3.40823100	2.62402700
H	8.45842000	3.69007900	1.46489000
H	8.64827200	2.09621800	2.19097400
C	-10.57011700	0.31638100	-2.43202400
H	-9.51845300	0.03747400	-2.51168900
H	-11.15644200	-0.59878800	-2.30525700
H	-10.86492900	0.75852100	-3.38811800
C	-9.19873400	2.95181500	1.79678800
H	-8.64821100	2.09614500	2.19120100
H	-8.45856300	3.69005500	1.46518000
H	-9.74739800	3.40802100	2.62437000
C	-10.57004400	-0.31652300	2.43205500
H	-9.51833900	-0.03779300	2.51187000
H	-11.15618000	0.59874600	2.30513800
H	-10.86509300	-0.75854800	3.38812600
C	-9.19880500	-2.95162400	-1.79701300
H	-8.64836900	-2.09589100	-2.19141500
H	-8.45855300	-3.68983500	-1.46552200
H	-9.74750600	-3.40780800	-2.62458200
C	-4.95614100	2.95054200	-0.56336100
H	-5.47454500	3.18702900	-1.49864100
H	-5.44535300	3.51846000	0.23508600
H	-3.93046300	3.31377800	-0.64975600
C	-4.95611300	-2.95047400	0.56341600
H	-5.47454700	-3.18698000	1.49867400
H	-5.44528100	-3.51839800	-0.23505400
H	-3.93043000	-3.31368900	0.64985000
C	4.95618800	2.95050400	-0.56352100
H	5.44538700	3.51845800	0.23490900
H	5.47459900	3.18695800	-1.49880500
H	3.93050700	3.31372800	-0.64994300
C	4.95617700	-2.95046200	0.56352900
H	5.44532300	-3.51842400	-0.23492800
H	5.47463700	-3.18692400	1.49878300
H	3.93049600	-3.31367100	0.65000900

Table S4. Z-matrix of the B3LYP/6-311G(d,p)-optimized geometry of **m-2**.

0 1

C	8.31825800	-0.73762200	0.02744300
C	8.14805900	0.67400000	-0.02657400
C	6.84658700	1.18291000	-0.02048200
C	5.68707000	0.38757500	0.00167700
C	5.86092000	-1.03833100	0.00339000
C	7.17413200	-1.53926100	0.02362500
C	4.35714100	1.00167500	0.01006700
C	3.32567000	0.11854500	0.00413700
C	3.51029200	-1.32045100	-0.00263500
C	4.71365800	-1.94198500	-0.00305400
C	1.83406700	-0.10588900	0.00211300
C	2.02855700	-1.49591800	-0.00277500
C	0.55419600	0.47996800	0.00049700
C	-0.55419200	-0.47999000	0.00045800
C	-0.30776000	-1.88197000	-0.00356600
C	0.97313800	-2.41310500	-0.00622700
C	0.30776600	1.88194800	-0.00343300
C	-0.97313300	2.41308300	-0.00605900
C	-2.02855100	1.49589600	-0.00267600
C	-1.83406200	0.10586700	0.00210100
C	-3.51028600	1.32043100	-0.00256200
C	-3.32566600	-0.11856600	0.00408400
C	-4.71365000	1.94196600	-0.00294500
C	-5.86091400	1.03831400	0.00339500
C	-5.68706500	-0.38759300	0.00154500
C	-4.35713800	-1.00169500	0.00989200
C	-7.17412500	1.53924300	0.02367900
C	-8.31825200	0.73760400	0.02740400
C	-8.14805500	-0.67401400	-0.02674700
C	-6.84658300	-1.18292500	-0.02068400
H	6.73672100	2.26171100	-0.03695200
H	7.31811000	-2.61442800	0.03951900
H	-1.16119000	-2.55033900	-0.00812600
H	1.13029800	-3.48693800	-0.01090300
H	1.16119700	2.55031700	-0.00795600
H	-1.13029200	3.48691600	-0.01066000
H	-7.31810400	2.61440800	0.03970200
H	-6.73671700	-2.26172500	-0.03722700
C	-9.64706400	1.43642200	0.13135100
C	-10.53098800	1.51597700	-0.96890100
C	-9.97892700	2.09758100	1.34121900
C	-11.74729800	2.19469800	-0.82475500
C	-11.20984100	2.75625700	1.44741800
C	-12.09735200	2.80021900	0.37691700
H	-12.42062600	2.25177300	-1.67611300
H	-11.46579000	3.24630200	2.38334100
H	-13.04799900	3.31685100	0.47412200
C	-9.27333500	-1.66785400	-0.13296000
C	-10.11545900	-1.95451300	0.96561400

C	-9.43720200	-2.38859300	-1.34326300
C	-11.13619500	-2.90196400	0.81952700
C	-10.47683200	-3.32013100	-1.45141100
C	-11.33059600	-3.57320500	-0.38248100
H	-11.77849700	-3.11681000	1.66966700
H	-10.60780000	-3.85668100	-2.38766700
H	-12.13164600	-4.30029800	-0.48120300
C	9.27332900	1.66785300	-0.13275900
C	10.11548500	1.95446800	0.96580300
C	9.43711400	2.38868700	-1.34301300
C	11.13618600	2.90196000	0.81973500
C	10.47671000	3.32026400	-1.45114600
C	11.33051800	3.57328500	-0.38223700
H	11.77851400	3.11677400	1.66986400
H	10.60761200	3.85689300	-2.38736600
H	12.13153800	4.30041300	-0.48094300
C	9.64707600	-1.43644700	0.13126700
C	10.53095500	-1.51587500	-0.96903000
C	9.97901400	-2.09770700	1.34106200
C	11.74728800	-2.19458200	-0.82500400
C	11.20995000	-2.75636200	1.44714200
C	12.09741000	-2.80020800	0.37659500
H	12.42057900	-2.25155700	-1.67639800
H	11.46595800	-3.24647800	2.38301100
H	13.04807600	-3.31682300	0.47370900
C	-10.19481600	0.92186200	-2.31608000
H	-9.12987400	1.01608100	-2.54449800
H	-10.44784400	-0.14206700	-2.35810900
H	-10.75679100	1.42607500	-3.10748200
C	-9.03584100	2.15248300	2.52618500
H	-8.46215600	1.23304900	2.65135900
H	-8.30826500	2.96574400	2.41683700
H	-9.59189300	2.33989100	3.44939800
C	-8.50631100	-2.21804000	-2.52686000
H	-8.16531500	-1.18913600	-2.65010100
H	-7.60774500	-2.83718000	-2.41745900
H	-9.00142300	-2.52989300	-3.45112300
C	-9.93261500	-1.29757100	2.31316800
H	-8.87640900	-1.13280400	2.54199900
H	-10.43436700	-0.32582300	2.35545200
H	-10.35705800	-1.92257000	3.10412400
C	9.03600800	-2.15272100	2.52608700
H	8.46232300	-1.23330400	2.65138400
H	8.30843300	-2.96597900	2.41671700
H	9.59212500	-2.34020600	3.44924500
C	10.19471600	-0.92162600	-2.31613200
H	9.12976400	-1.01582800	-2.54450900
H	10.44773600	0.14230900	-2.35806800
H	10.75665500	-1.42575600	-3.10761200
C	9.93270300	1.29745200	2.31332900
H	8.87651100	1.13263700	2.54218500
H	10.43449100	0.32572100	2.35554700

H	10.35714900	1.92242600	3.10430400
C	8.50613900	2.21820800	-2.52655400
H	8.16519400	1.18929700	-2.64987500
H	7.60754300	2.83728000	-2.41701500
H	9.00115900	2.53018900	-3.45082300
C	4.87537500	-3.43860700	-0.00793300
H	5.43790800	-3.78256700	-0.88386200
H	5.41529600	-3.79135500	0.87890300
H	3.90072400	-3.93204900	-0.02222200
C	4.20298000	2.49881600	0.02349200
H	4.64118300	2.95867000	-0.87028200
H	3.15164700	2.78487300	0.06140200
H	4.70025300	2.94649000	0.89170900
C	-4.20298000	-2.49883700	0.02316200
H	-4.64123600	-2.95860300	-0.87063100
H	-3.15164600	-2.78490100	0.06097700
H	-4.70020600	-2.94659600	0.89136500
C	-4.87536600	3.43858800	-0.00769100
H	-5.43788400	3.78263000	-0.88359700
H	-5.41530200	3.79125600	0.87916900
H	-3.90071400	3.93203100	-0.02191700

Table S5. Z-matrix of the UB3LYP/6-311G(d,p)-optimized geometry of the radical cation of **m-2**.
1 2

C	-8.28798800	-0.74338100	-0.03028600
C	-8.11650800	0.67855200	0.02764900
C	-6.81981400	1.18890100	0.02455000
C	-5.65666100	0.39444200	0.00318900
C	-5.83161200	-1.04104200	-0.00145700
C	-7.14852300	-1.54287700	-0.02435500
C	-4.33977200	1.01431300	-0.00570900
C	-3.30344200	0.12636400	0.00172800
C	-3.48877300	-1.31697300	0.00996900
C	-4.69924100	-1.94498000	0.00825000
C	-1.82866400	-0.08687800	0.00584100
C	-2.03012900	-1.50295500	0.01138000
C	-0.55185200	0.48455700	0.00861900
C	0.55187700	-0.48455700	0.00864100
C	0.29892200	-1.88714000	0.01344000
C	-0.98373700	-2.42252900	0.01569000
C	-0.29889700	1.88714100	0.01335500
C	0.98376200	2.42253000	0.01557800
C	2.03015300	1.50295500	0.01130800
C	1.82868900	0.08687900	0.00583800
C	3.48879800	1.31697300	0.00990400
C	3.30346600	-0.12636400	0.00173600
C	4.69926500	1.94498000	0.00815000
C	5.83163600	1.04104100	-0.00151500
C	5.65668400	-0.39444200	0.00321000
C	4.33979600	-1.01431400	-0.00565100
C	7.14854600	1.54287300	-0.02444400
C	8.28801400	0.74338100	-0.03033300

C	8.11653300	-0.67855400	0.02767200
C	6.81983700	-1.18889800	0.02461000
H	-6.71337100	2.26718900	0.03962800
H	-7.29274100	-2.61716300	-0.03810000
H	1.15066200	-2.55720200	0.01894500
H	-1.14131800	-3.49515600	0.02094300
H	-1.15063800	2.55720200	0.01883400
H	1.14134300	3.49515700	0.02078200
H	7.29276300	2.61715900	-0.03824400
H	6.71339300	-2.26718600	0.03974900
C	9.61712000	1.43686900	-0.13073800
C	10.50487900	1.48988500	0.96842700
C	9.94118400	2.11659800	-1.33282900
C	11.72201500	2.16678800	0.82789500
C	11.17534700	2.76859600	-1.43343700
C	12.06766700	2.78969400	-0.36606000
H	12.39984100	2.20867600	1.67594200
H	11.43102800	3.27139600	-2.36207400
H	13.01990500	3.30299300	-0.45971900
C	9.24388600	-1.66675500	0.12560600
C	10.09270000	-1.92686000	-0.97491600
C	9.40012800	-2.40471300	1.32704800
C	11.11549900	-2.87247000	-0.83661300
C	10.44543500	-3.32983400	1.42536700
C	11.30586900	-3.56045700	0.35644500
H	11.76314400	-3.07243200	-1.68571000
H	10.57701500	-3.87891000	2.35368000
H	12.11016400	-4.28409800	0.44843000
C	-9.24387700	1.66674000	0.12551900
C	-10.09271100	1.92671500	-0.97501700
C	-9.40015900	2.40476500	1.32691300
C	-11.11554200	2.87230100	-0.83678800
C	-10.44550000	3.32985800	1.42516000
C	-11.30593400	3.56037700	0.35621600
H	-11.76320300	3.07216600	-1.68589500
H	-10.57711000	3.87898700	2.35343700
H	-12.11025800	4.28399200	0.44814600
C	-9.61710500	-1.43685100	-0.13064300
C	-10.50488900	-1.48969400	0.96850900
C	-9.94117900	-2.11669000	-1.33266700
C	-11.72204600	-2.16657100	0.82803700
C	-11.17536200	-2.76866000	-1.43321800
C	-12.06769700	-2.78960900	-0.36585100
H	-12.39989300	-2.20832700	1.67607300
H	-11.43104900	-3.27155200	-2.36180500
H	-13.01995300	-3.30288200	-0.45946600
C	10.17518400	0.87828000	2.30970500
H	9.10845200	0.94679500	2.53967900
H	10.45572700	-0.17880800	2.34713100
H	10.72138100	1.39084300	3.10602100
C	8.99714100	2.19328000	-2.51634000
H	8.41160800	1.28269100	-2.65405200

H	8.28384800	3.01909500	-2.40412900
H	9.55584900	2.37907800	-3.43749200
C	8.46777900	-2.25536400	2.51288300
H	8.11715500	-1.23141600	2.65275400
H	7.57757400	-2.88676700	2.40218900
H	8.96827500	-2.57025100	3.43241900
C	9.91562700	-1.25341100	-2.31540100
H	8.86323000	-1.06202600	-2.54166200
H	10.44313200	-0.29539500	-2.35426900
H	10.31917900	-1.88250700	-3.11327700
C	-8.99712400	-2.19352900	-2.51615900
H	-8.41152500	-1.28299200	-2.65393300
H	-8.28389200	-3.01938600	-2.40387000
H	-9.55583000	-2.37936100	-3.43730500
C	-10.17520800	-0.87789500	2.30970300
H	-9.10848800	-0.94643100	2.53972500
H	-10.45569300	0.17921600	2.34695100
H	-10.72146200	-1.39030000	3.10608200
C	-9.91564700	1.25311500	-2.31542700
H	-8.86324300	1.06179900	-2.54172100
H	-10.44306200	0.29504200	-2.35414500
H	-10.31930100	1.88207100	-3.11336100
C	-8.46783000	2.25551300	2.51277700
H	-8.11714900	1.23159100	2.65269500
H	-7.57766200	2.88696600	2.40207800
H	-8.96836500	2.57040600	3.43229000
C	-4.85399000	-3.44059800	0.01360800
H	-5.41933600	-3.78086200	0.88789500
H	-5.39022600	-3.79068900	-0.87517700
H	-3.87986900	-3.93348800	0.03189600
C	-4.18504100	2.50994900	-0.02122700
H	-4.61714700	2.96643700	0.87601000
H	-3.13538600	2.79929100	-0.06788000
H	-4.68955900	2.95350100	-0.88606300
C	4.18506500	-2.50995100	-0.02108400
H	4.61719600	-2.96638900	0.87616700
H	3.13540900	-2.79929700	-0.06769200
H	4.68956200	-2.95354900	-0.88590900
C	4.85401500	3.44059800	0.01343400
H	5.41936600	3.78090400	0.88770200
H	5.39024500	3.79064600	-0.87537100
H	3.87989400	3.93348900	0.03170500

Table S6. Z-matrix of the (U)B3LYP/6-311G(d,p)-optimized geometry of the dication of **m-2**.

A) B3LYP/6-311G(D,P) CLOSED-SHELL STATE OF THE DICATION OF M-2.

2 1

C	-8.27209600	0.75138800	0.02206500
C	-8.09939600	-0.68288500	-0.01798900
C	-6.80172200	-1.19505200	-0.03501300
C	-5.63983400	-0.40421700	-0.01493300
C	-5.81543000	1.04364000	0.00524100
C	-7.13225400	1.54740400	0.02613900
C	-4.32906800	-1.02946500	-0.00365400
C	-3.29088200	-0.14004600	-0.00668600
C	-3.47711400	1.31148600	-0.01469400
C	-4.69215800	1.94589600	-0.00877700
C	-1.82739900	0.07088600	-0.01064200
C	-2.03648200	1.50079700	-0.01522200
C	-0.54879300	-0.48883300	-0.01279000
C	0.54877800	0.48884900	-0.01286400
C	0.28675700	1.89144600	-0.01546800
C	-0.99456800	2.42643800	-0.01693200
C	-0.28677300	-1.89142900	-0.01519300
C	0.99455300	-2.42642200	-0.01658700
C	2.03646600	-1.50078000	-0.01501700
C	1.82738300	-0.07086900	-0.01064500
C	3.47709800	-1.31146900	-0.01452400
C	3.29086700	0.14006400	-0.00673100
C	4.69214200	-1.94587900	-0.00851900
C	5.81541400	-1.04362100	0.00535500
C	5.63981900	0.40423400	-0.01502900
C	4.32905300	1.02948300	-0.00383900
C	7.13223700	-1.54738300	0.02632600
C	8.27207800	-0.75136600	0.02213200
C	8.09938000	0.68290100	-0.01812300
C	6.80170700	1.19506700	-0.03521400
H	-6.69675000	-2.27261400	-0.03507600
H	-7.27295900	2.62135900	0.02066400
H	1.13575000	2.56524400	-0.01881900
H	-1.15464800	3.49827100	-0.02025300
H	-1.13576500	-2.56522900	-0.01844400
H	1.15463300	-3.49825500	-0.01975200
H	7.27294300	-2.62133900	0.02102100
H	6.69673100	2.27262800	-0.03540900
C	9.60175100	-1.43006900	0.05879600
C	10.50399500	-1.33013700	-1.03365800
C	9.93032000	-2.24494100	1.17889700
C	11.73610500	-1.98658700	-0.95621400
C	11.18170400	-2.86370400	1.21631200
C	12.08613600	-2.73349000	0.16287800
H	12.42050800	-1.91946000	-1.79683800
H	11.44978300	-3.45832000	2.08469100
H	13.05097200	-3.22859800	0.20951600
C	9.22961900	1.65468300	-0.04711600

C	10.13256100	1.76249100	1.04557900
C	9.35804000	2.53081600	-1.16378600
C	11.17536900	2.69059000	0.97241200
C	10.43008600	3.42394500	-1.19718400
C	11.34006900	3.50426700	-0.14278300
H	11.85640000	2.78216800	1.81342400
H	10.55401400	4.06645600	-2.06393700
H	12.16230500	4.21154000	-0.18643900
C	-9.22961800	-1.65468800	-0.04687800
C	-10.13257000	-1.76239300	1.04582000
C	-9.35796400	-2.53099000	-1.16342000
C	-11.17533500	-2.69054600	0.97276000
C	-10.42996000	-3.42418500	-1.19670900
C	-11.33996700	-3.50439500	-0.14232200
H	-11.85638000	-2.78203800	1.81376900
H	-10.55382600	-4.06683900	-2.06336600
H	-12.16216200	-4.21172000	-0.18589100
C	-9.60177100	1.43009200	0.05862500
C	-10.50399400	1.33003500	-1.03383300
C	-9.93036100	2.24509000	1.17862800
C	-11.73610400	1.98649800	-0.95649000
C	-11.18174900	2.86385000	1.21595300
C	-12.08616000	2.73352000	0.16251500
H	-12.42048600	1.91928300	-1.79712300
H	-11.44984800	3.45855600	2.08426300
H	-13.05099800	3.22863100	0.20908100
C	10.16874800	-0.61166600	-2.32049100
H	9.09265800	-0.52604400	-2.48390500
H	10.59498300	0.39598800	-2.34043300
H	10.58717500	-1.15656500	-3.17117000
C	8.99773600	-2.47177700	2.35208600
H	8.35112800	-1.61772400	2.56078400
H	8.34745100	-3.33869500	2.18230100
H	9.57328600	-2.68465900	3.25629400
C	8.40313400	2.53266900	-2.34090200
H	7.98800700	1.54755600	-2.56046500
H	7.55888800	3.21170800	-2.16941700
H	8.91337100	2.88829700	-3.23936900
C	9.97487600	0.98213800	2.32995400
H	8.95143900	0.63711200	2.48789400
H	10.63314700	0.10839700	2.35035000
H	10.24467100	1.61103700	3.18288000
C	-8.99780000	2.47205000	2.35180900
H	-8.35131400	1.61795200	2.56071400
H	-8.34738700	3.33884200	2.18187300
H	-9.57336100	2.68519700	3.25594700
C	-10.16872600	0.61141700	-2.32057700
H	-9.09263300	0.52577300	-2.48396500
H	-10.59496100	-0.39623900	-2.34040400
H	-10.58714200	1.15621400	-3.17132700
C	-9.97492000	-0.98186800	2.33009600
H	-8.95146100	-0.63692600	2.48807500

H	-10.63310000	-0.10805200	2.35030500
H	-10.24485500	-1.61061300	3.18309100
C	-8.40301700	-2.53297800	-2.34050300
H	-7.98782100	-1.54790700	-2.56011400
H	-7.55882300	-3.21206300	-2.16894000
H	-8.91324500	-2.88863000	-3.23896600
C	-4.84007300	3.44115700	-0.01005300
H	-5.40380400	3.78426400	-0.88399900
H	-5.37415900	3.78945400	0.88022700
H	-3.86520300	3.93143600	-0.02773200
C	-4.17667600	-2.52459500	0.00471900
H	-4.61618100	-2.97545300	-0.89126600
H	-3.12764100	-2.81660300	0.04068600
H	-4.67331300	-2.96999800	0.87290200
C	4.17666000	2.52461400	0.00430900
H	4.61618000	2.97533800	-0.89173600
H	3.12762500	2.81662700	0.04021400
H	4.67328100	2.97014700	0.87243500
C	4.84005600	-3.44114000	-0.00956700
H	5.40378400	-3.78438100	-0.88346100
H	5.37414400	-3.78929900	0.88076600
H	3.86518600	-3.93142100	-0.02716700

B) UB3LYP/6-311G(D,P) OPEN-SHELL BROKEN SYMMETRY - DICATION OF M-2.

2 1

C	-8.27423100	0.75245700	0.02116100
C	-8.10172100	-0.68182500	-0.01690700
C	-6.80415300	-1.19399100	-0.03536500
C	-5.64309600	-0.40210200	-0.01576600
C	-5.81870800	1.04530900	0.00440600
C	-7.13416200	1.54943400	0.02684200
C	-4.33080900	-1.02650400	-0.00481400
C	-3.29388500	-0.13703800	-0.00891100
C	-3.48038000	1.31446400	-0.01697100
C	-4.69287900	1.94825500	-0.01080300
C	-1.82846000	0.07628700	-0.01360200
C	-2.03485100	1.50090100	-0.01813600
C	-0.54855000	-0.48746300	-0.01610200
C	0.54854100	0.48752400	-0.01615700
C	0.28935400	1.89050300	-0.01905800
C	-0.99151600	2.42564200	-0.02039500
C	-0.28936300	-1.89044200	-0.01892200
C	0.99150700	-2.42558200	-0.02027500
C	2.03484200	-1.50084000	-0.01812100
C	1.82845100	-0.07622600	-0.01367500
C	3.48037000	-1.31440300	-0.01702700
C	3.29387700	0.13709900	-0.00905900
C	4.69286900	-1.94819500	-0.01085900
C	5.81869800	-1.04524800	0.00424000
C	5.64308700	0.40216100	-0.01602300

C	4.33080100	1.02656500	-0.00506000
C	7.13415200	-1.54937100	0.02671200
C	8.27422000	-0.75239500	0.02093800
C	8.10171400	0.68188800	-0.01721500
C	6.80414400	1.19405100	-0.03566300
H	-6.69807900	-2.27143300	-0.03442800
H	-7.27452100	2.62339100	0.02126700
H	1.13902800	2.56325000	-0.02281800
H	-1.15095500	3.49764400	-0.02392200
H	-1.13903700	-2.56319000	-0.02260400
H	1.15094600	-3.49758400	-0.02373700
H	7.27451400	-2.62333000	0.02127200
H	6.69806300	2.27149000	-0.03470600
C	9.60491500	-1.42674800	0.05382200
C	10.51106800	-1.31139100	-1.03587200
C	9.93556500	-2.25100600	1.16727000
C	11.74852600	-1.95798900	-0.95956300
C	11.19150500	-2.85921600	1.20323500
C	12.09982800	-2.71172000	0.15398000
H	12.43515300	-1.87851300	-1.79726600
H	11.46218300	-3.45881100	2.06734100
H	13.06828100	-3.19972300	0.20045300
C	9.23443500	1.64996600	-0.04056200
C	10.14078300	1.74518700	1.05211400
C	9.36603900	2.53426200	-1.15084300
C	11.19129400	2.66506300	0.98257500
C	10.44465400	3.41889800	-1.18017500
C	11.35898500	3.48466800	-0.12725200
H	11.87482900	2.74591600	1.82264200
H	10.57204800	4.06642900	-2.04266400
H	12.18631400	4.18610900	-0.16873800
C	-9.23442300	-1.64991900	-0.04039000
C	-10.14097000	-1.74512800	1.05212100
C	-9.36570700	-2.53433500	-1.15059900
C	-11.19139400	-2.66509300	0.98244300
C	-10.44423300	-3.41907600	-1.18007700
C	-11.35878800	-3.48481300	-0.12734700
H	-11.87509000	-2.74593400	1.82237900
H	-10.57136700	-4.06672500	-2.04251600
H	-12.18604100	-4.18633600	-0.16893500
C	-9.60494200	1.42679000	0.05385400
C	-10.51098000	1.31125000	-1.03591400
C	-9.93579100	2.25109700	1.16721500
C	-11.74849200	1.95776700	-0.95980500
C	-11.19178500	2.85920700	1.20298600
C	-12.09997100	2.71157100	0.15363200
H	-12.43502700	1.87815100	-1.79757000
H	-11.46262200	3.45882500	2.06702600
H	-13.06847000	3.19949600	0.19996000
C	10.17286300	-0.59243900	-2.32147300
H	9.09721500	-0.48541800	-2.47179300
H	10.62070200	0.40555600	-2.35240300

H	10.56922700	-1.15062000	-3.17431500
C	9.00180100	-2.49510600	2.33587500
H	8.34948100	-1.64718900	2.55141900
H	8.35715600	-3.36397100	2.15548400
H	9.57663700	-2.71380800	3.23912000
C	8.40881600	2.55192900	-2.32578800
H	7.98558100	1.57171900	-2.55151900
H	7.57011500	3.23576800	-2.14685200
H	8.91964000	2.91067200	-3.22266800
C	9.97673000	0.96389900	2.33503200
H	8.95815500	0.59956800	2.47772900
H	10.65234600	0.10381800	2.36833300
H	10.22084500	1.60002500	3.19053700
C	-9.00224600	2.49528800	2.33597600
H	-8.34999300	1.64737400	2.55173700
H	-8.35754100	3.36411700	2.15563300
H	-9.57725600	2.71409600	3.23908600
C	-10.17260800	0.59212300	-2.32137200
H	-9.09693600	0.48520300	-2.47160200
H	-10.62032300	-0.40593200	-2.35215700
H	-10.56898400	1.15011000	-3.17433500
C	-9.97720700	-0.96375700	2.33502700
H	-8.95867000	-0.59939900	2.47792200
H	-10.65284600	-0.10368500	2.36813100
H	-10.22149400	-1.59983300	3.19051900
C	-8.40814100	-2.55209100	-2.32526400
H	-7.98480700	-1.57190400	-2.55091200
H	-7.56951400	-3.23594700	-2.14603800
H	-8.91870800	-2.91086000	-3.22228000
C	-4.84252500	3.44360800	-0.01228400
H	-5.40630000	3.78671200	-0.88625500
H	-5.37611300	3.79219200	0.87825700
H	-3.86780400	3.93425000	-0.03029800
C	-4.17830000	-2.52180900	0.00401300
H	-4.61730500	-2.97342300	-0.89191300
H	-3.12912500	-2.81317800	0.04015300
H	-4.67444300	-2.96739600	0.87244600
C	4.17829100	2.52187000	0.00368300
H	4.61727000	2.97343100	-0.89228300
H	3.12911700	2.81324100	0.03983300
H	4.67445900	2.96750900	0.87207600
C	4.84251600	-3.44354800	-0.01222400
H	5.40625800	-3.78672300	-0.88618800
H	5.37613900	-3.79205700	0.87832600
H	3.86779400	-3.93419200	-0.03016000

Table S7. Normal mode description of the more intense Raman wavenumbers (b3lyp-6-311g**) of the [3]naphthylene **m-1** model molecule (which is **1** without methyl nor xylyl groups) in the 1800–800 cm^{-1} spectral region. To clarify, similar vibrational contributions were accumulated in a single term. The meaning of the symbols is: ν(stretching mode), δ(in-plane bending mode), CBD(vibration of the cyclobutadienoid moieties), NAP(vibrations of the naphthalene moieties), CH(carbon–hydrogen bond).

Experimental wavenumber (cm^{-1})	Experimental Raman intensity	Calculated wavenumber (cm^{-1})	Calculated Raman activity	Potential energy distribution (contributions greater than 10%)
1700	strong	1730	17410	55ν(NAP), 30(CBD)
1607	weak	1615	59	72(NAP), 14ν(CBD)
1538	weak	1555	2335	90(NAP)
1448	strong	1468	10281	82(NAP), 20δ(CH)
1424	weak	1436	570	55δ(CH), 21ν(CBD), 19ν(NAP)
1424	weak	1408	3246	58δ(CH), 25ν(CBD)
1375	weak	1358	2064	100ν(NAP)
1355	strong	1354	10044	100ν(NAP), 12δ(CH)
1215	weak	1208	7	44ν(NAP), 43δ(CH)
1167	weak	1174	235	38ν(NAP), 37δ(CH), 20δ(NAP)
1082	medium	1118	3281	78ν(CBD), 16δ(CH)
1032	weak	1052	405	65ν(NAP), 14δ(CH)
1026	weak	1034	2	43ν(CBD), 31δ(CH)
841	weak	855	36	32ν(CBD), 29ν(NAP),

Figure S2. Full vibrational normal mode associated with the theoretical Raman band of **m-1** at 1730 cm^{-1}. The size of the spheres is proportional to the vibrational amplitudes (blue is for the elongation and yellow for the compression motion respectively)

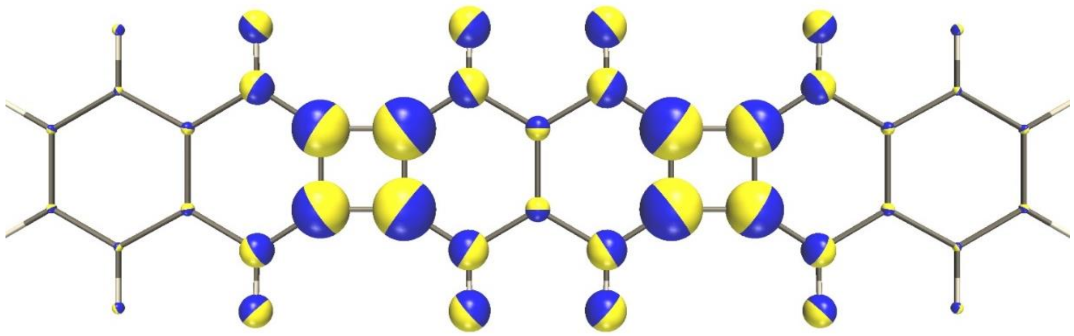

Figure S3. Theoretical Raman spectrum (dotted line) of an unsubstituted model of **2** (denoted as **m-2)** which is **2** without methyl nor xylyl groups compared with the experimental (solid line) FT-Raman spectrum of **2** in the solid state at room temperature.

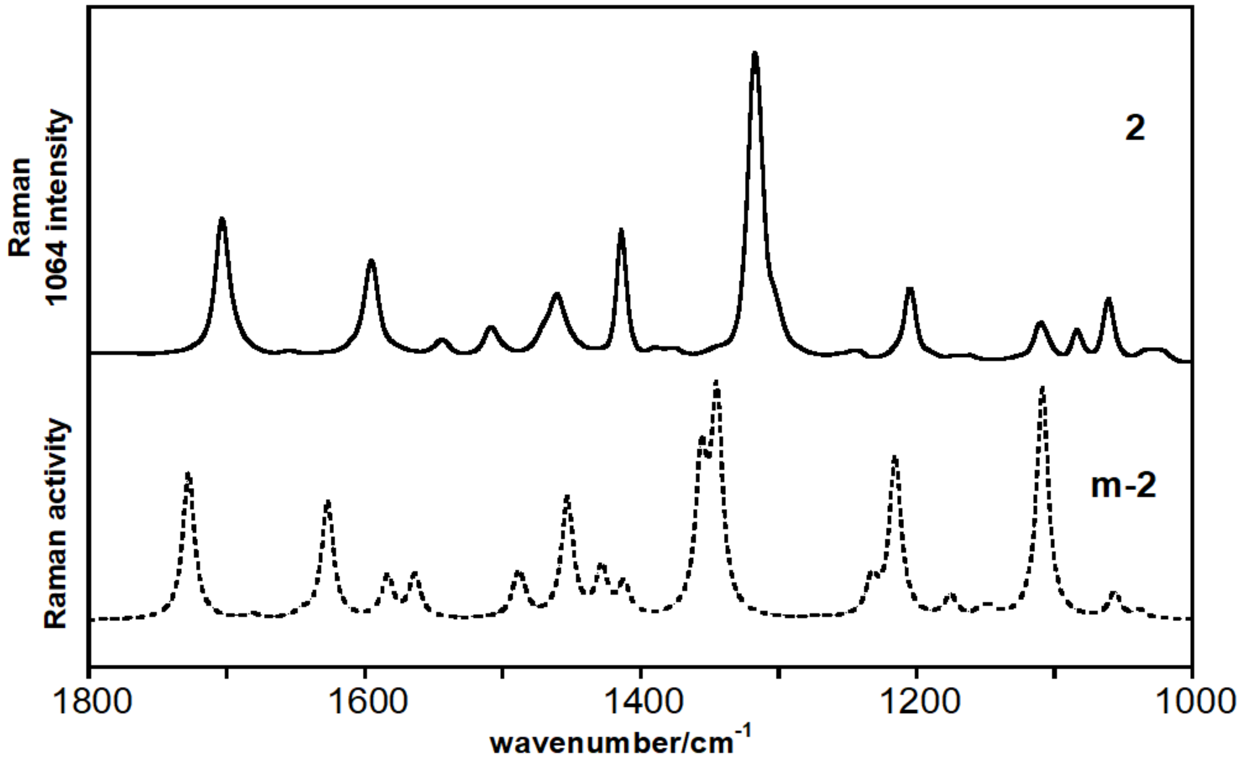

Force field calculations

Quadratic (harmonic) Cartesian force constants were evaluated within the same theoretical scheme used for the geometry optimizations. The Cartesian force constants were transformed into a set of non-redundant locally symmetrised internal coordinates following the Wilson procedure. [1] This method starts with the definition of a full space of displacement internal coordinates ***R***, where ***R*** can be built from as full set of bond stretching, angle bending or torsional vibrations. [2-4] For molecules, local (three branches) and cyclic redundancies usually occur and can be completely removed by defining a new set of 3N-6 symmetry or group coordinates ***S***, orthogonal to the redundancy conditions to remove systematically all redundancies occurring in our system. [5] Molecular symmetry allows for factorising the force constant matrix into one square matrix for each symmetry species of the punctual group to which the molecule belongs, being null all the interaction constants between coordinates of different symmetry. Here, force field calculation was restricted to the totally symmetric species (A_g in both the D_{2h} and the C_{2h} groups, to which the **m-1** and the **m-2** model molecules belong, respectively).

In matrix notation the linear transformations between coordinates are $\boldsymbol{R = Bx}$ and $\boldsymbol{S = UR}$. Then the potential energy in Cartesian coordinates, $\boldsymbol{2V = x^t F_x x}$, can be re-expressed in terms of the $\boldsymbol{S}$ coordinates as:

$$\boldsymbol{2V = S^t U^{-1t} B^{-1t} F_x B^{-1} U^{-1} S}$$

Then, the equation for the vibrational problem of any finite molecule is:

$$\boldsymbol{G_S F_S L_S = L_S \Lambda}$$

where $\boldsymbol{G_S}$ is the kinetic energy matrix, which contains information about the atoms which constitute the molecule and their equilibrium positions, $\boldsymbol{F_S}$ is the matrix of the locally symmetrized force constants, $\boldsymbol{L_S}$ is the matrix of the vibrational amplitudes and $\boldsymbol{\Lambda}$ is the diagonal eigenvalue matrix. The normal frequencies λ_i, in cm^{-1}, can be obtained from eigenvalues, $\boldsymbol{\nu_i}$, through the relation $\boldsymbol{\nu_i = (\lambda_i / 4\pi^2 c^2)^{1/2}}$.

[1] E.B. Wilson, J.C. Decius, P.C. Cross, Molecular Vibrations, McGraw-Hill, New York, 1955.

[2] S. Califano, Vibrational States, Wiley, New York, 1976.

[3] L.A. Grivob, W.J. Orville-Thomas, Theory and Methods of Calculations of Molecular Spectra, Wiley, New York, 1988.

[4] G. Zerbi, Vibrational Intensities in Infrared and Raman Spectroscopy, Elsevier, Amsterdam, 1984.

[5] P. Pulay, G. Fogarasi, G. Pongor, J.E. Boggs, J. Am. Chem. Soc. 102 (1979) 2550.

Figure S4. Theoretical Raman spectra ((U)b3lyp-6-311g**) of neutral (black line) and oxidized species of **m-1**, radical cation (blue line) and dication (red line).

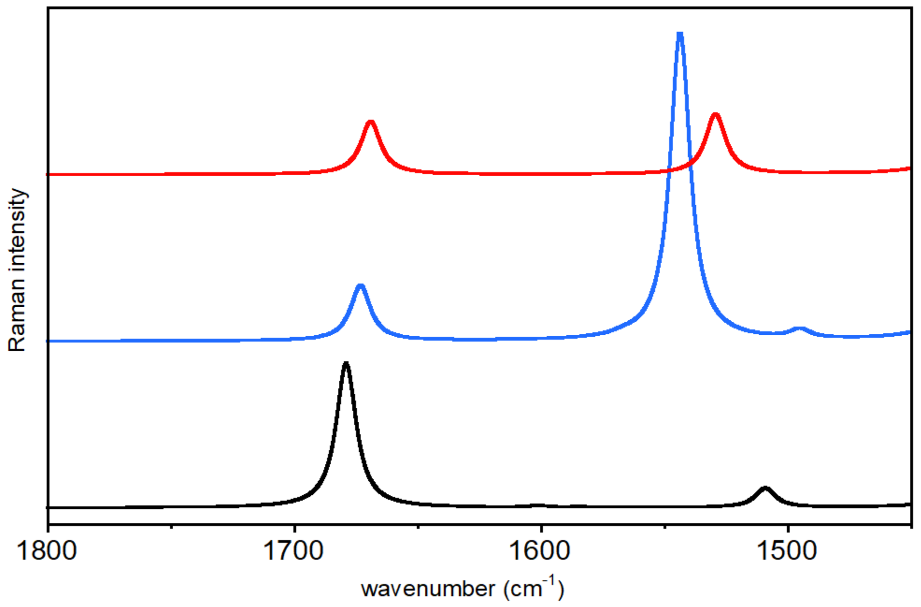

Figure S5. Theoretical Raman spectra ((U)b3lyp-6-311g**) of neutral (black line) and oxidized species of **m-2**, radical cation (blue line) and dication (red line).

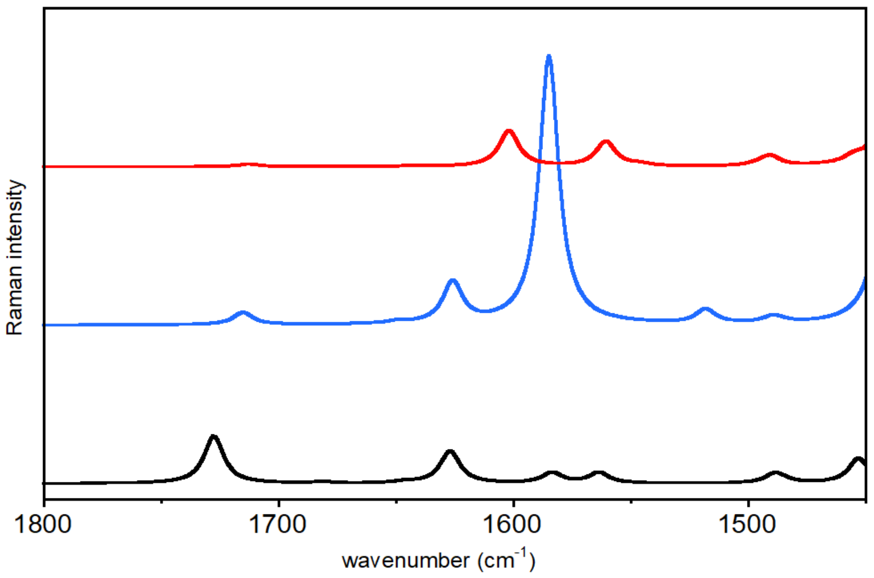

Figure S6. ACID plots of neutral (top) and radical cation (bottom) **m-1** at an isosurface value of 0.04. Current density vectors (green arrows with red arrowheads) are plotted onto the isosurface. The level of theory for the neutral species was b3lyp/6-311g(d), while for the radical cation was ub3lyp/6-311g(d).

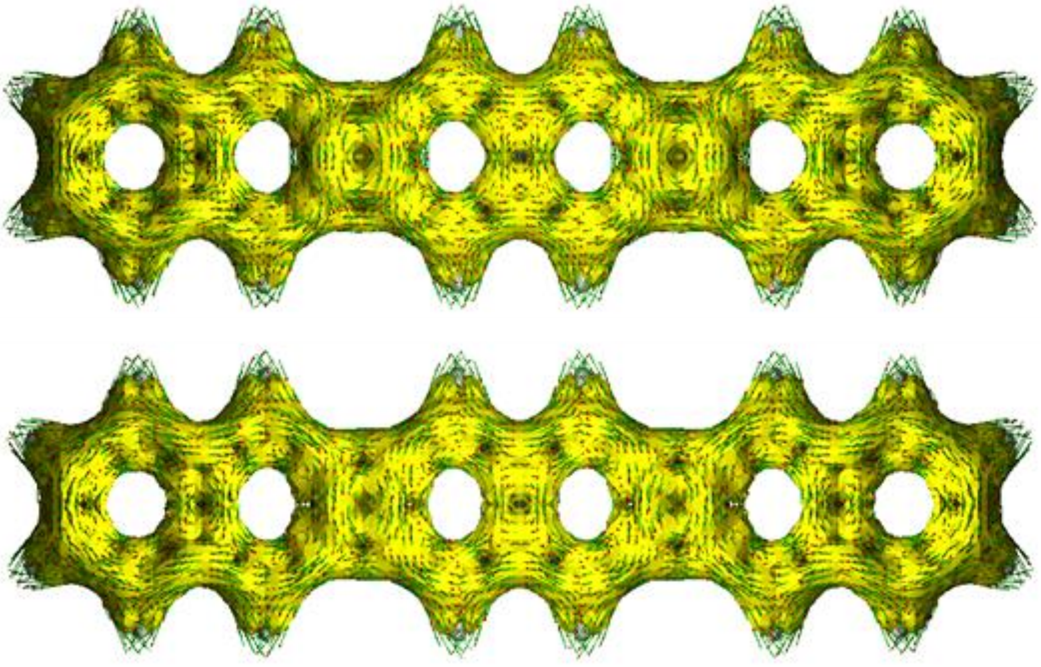

Figure S7. ACID plots of neutral (top) and radical cation (bottom) **m-2** at an isosurface value of 0.04. Current density vectors (green arrows with red arrowheads) are plotted onto the isosurface. The level of theory for the neutral species was b3lyp/6-311g(d), while for the radical cation was ub3lyp/6-311g(d).

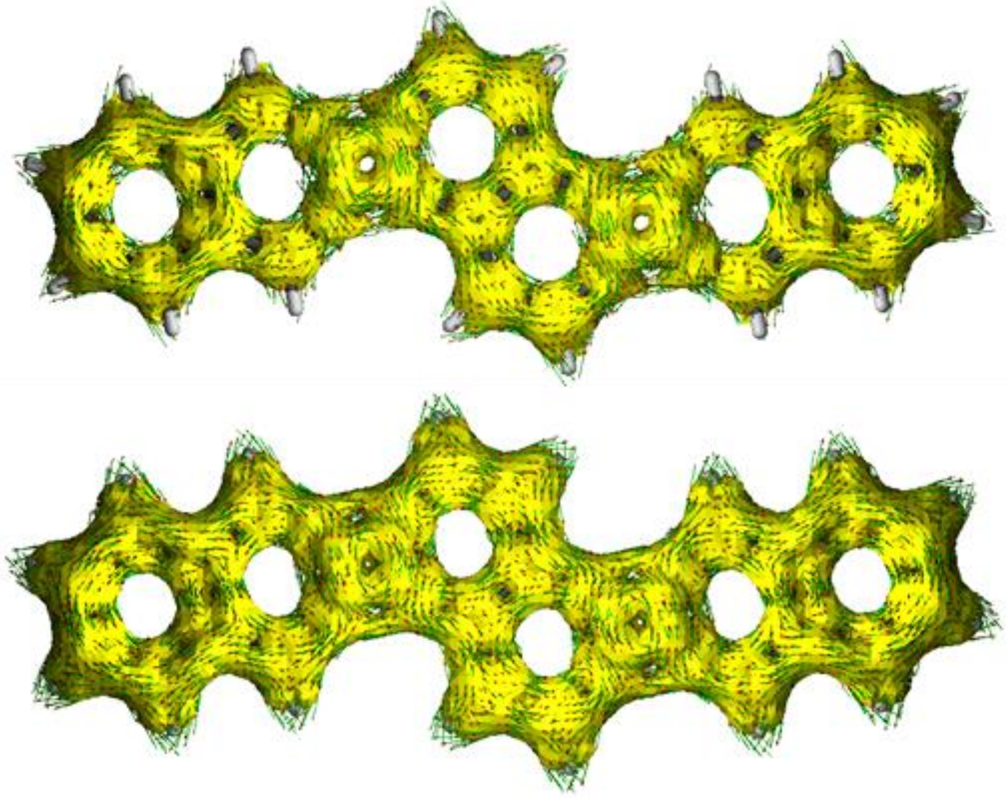

Supplement: File 1 — Chemical structures of m-1 and m-2, vibrational assignment of m-1, the 1730 cm−1 normal mode, theoretical Raman spectrum of m-1, details of the force field calculations, theoretical Raman spectra of m-1 and m-2, and ACID plots of m-1 and m-2. [file Beilstein_J_Org_Chem-21-277-s001.pdf]
